# Phylogeny and molecular signatures (conserved proteins and indels) that are specific for the Bacteroidetes and Chlorobi species

**DOI:** 10.1186/1471-2148-7-71

**Published:** 2007-05-08

**Authors:** Radhey S Gupta, Emily Lorenzini

**Affiliations:** 1 Department of Biochemistry and Biomedical Science, McMaster University, Hamilton, L8N3Z5, Canada

## Abstract

**Background:**

The *Bacteroidetes *and *Chlorobi *species constitute two main groups of the *Bacteria *that are closely related in phylogenetic trees. The *Bacteroidetes *species are widely distributed and include many important periodontal pathogens. In contrast, all *Chlorobi *are anoxygenic obligate photoautotrophs. Very few (or no) biochemical or molecular characteristics are known that are distinctive characteristics of these bacteria, or are commonly shared by them.

**Results:**

Systematic blast searches were performed on each open reading frame in the genomes of *Porphyromonas gingivalis *W83, *Bacteroides fragilis *YCH46, *B. thetaiotaomicron *VPI-5482, *Gramella forsetii KT0803, Chlorobium luteolum *(formerly *Pelodictyon luteolum*) DSM 273 and *Chlorobaculum tepidum *(formerly *Chlorobium tepidum*) TLS to search for proteins that are uniquely present in either all or certain subgroups of *Bacteroidetes *and *Chlorobi*. These studies have identified > 600 proteins for which homologues are not found in other organisms. This includes 27 and 51 proteins that are specific for most of the sequenced *Bacteroidetes *and *Chlorobi *genomes, respectively; 52 and 38 proteins that are limited to species from the *Bacteroidales *and *Flavobacteriales *orders, respectively, and 5 proteins that are common to species from these two orders; 185 proteins that are specific for the *Bacteroides *genus. Additionally, 6 proteins that are uniquely shared by species from the *Bacteroidetes *and *Chlorobi *phyla (one of them also present in the *Fibrobacteres*) have also been identified. This work also describes two large conserved inserts in DNA polymerase III (DnaE) and alanyl-tRNA synthetase that are distinctive characteristics of the *Chlorobi *species and a 3 aa deletion in ClpB chaperone that is mainly found in various *Bacteroidales, Flavobacteriales *and *Flexebacteraceae*, but generally not found in the homologs from other organisms. Phylogenetic analyses of the *Bacteroidetes *and *Chlorobi *species is also reported based on concatenated sequences for 12 conserved proteins by different methods including the character compatibility (or clique) approach. The placement of *Salinibacter ruber *with other *Bacteroidetes *species was not resolved by other phylogenetic methods, but this affiliation was strongly supported by the character compatibility approach.

**Conclusion:**

The molecular signatures described here provide novel tools for identifying and circumscribing species from the *Bacteroidetes *and *Chlorobi *phyla as well as some of their main groups in clear terms. These results also provide strong evidence that species from these two phyla (and also possibly *Fibrobacteres*) are specifically related to each other and they form a single superphylum. Functional studies on these proteins and indels should aid in the discovery of novel biochemical and physiological characteristics that are unique to these groups of bacteria.

## Background

The *Bacteroidetes *and *Chlorobi *presently comprise two of the main phyla within the *Bacteria *[1-3]. The bacteria from the *Bacteroidetes *phylum (previously known as the Cytophaga-Flavobacteria-Bacteroides (CFB) group) exhibit a *potpourri *of phenotypes including gliding behavior and their ability to digest and grow on a variety of complex substrates such as cellulose, chitin and agar [4-8]. They inhabit diverse habitats including the oral cavity of humans, the gastrointestinal tracts of mammals, saturated thalassic brines, soil and fresh water [9-13]. The *Bacteroides *species such as *B. thetaiotaomicron *and *B. fragilis *are among the dominant microbes in the large intestine of human and other animals [14,15]. These bacteria in the human colon are also important opportunistic pathogens and they are involved in causing abscesses and soft tissue infections of the gastrointestinal tract, as well as diarrheal diseases [15-18]. Other bacteroidetes species, such as *Porphyromonas gingivalis *and *Prevotella intermedia*, are major causative agents in the initiation and progression of periodontal disease in humans [12,19,20].

In contrast to wide distribution of *Bacteroidetes *species in diverse habitats, bacteria from the phylum *Chlorobi *occupy a narrow environmental niche mainly consisting of anoxic aquatic settings in stratified lakes (chemocline regions), where sunlight is able to penetrate [21-24]. Some of these bacteria also exist as epibionts in phototrophic consortiums with other bacteria, particularly β-proteobacteria [21,25]. The *Chlorobi*, which are also commonly known as Green Sulfur bacteria, are all anoxygenic obligate photoautotrophs, which obtain electrons for anaerobic photosynthesis from hydrogen sulfide [22,23,26]. Although the *Bacteroidetes *and *Chlorobi *are presently recognized as two distinct phyla [1,3], these two groups are closely related in phylogenetic trees based on 16S rRNA as well other gene sequences [27-30]. Conserved indels (i.e. inserts or deletions) in a number of widely distributed proteins (viz. FtsK, UvrB and ATP synthase α subunit), that are uniquely present in species from these two groups, also strongly indicate that these two groups of species shared a common ancestor exclusive of all other bacteria [30].

The species from the *Bacteroidetes *and *Chlorobi *phyla are presently distinguished from other bacteria primarily on the basis of their branching in phylogenetic trees [2,3,27]. We have previously described a 4 aa conserved insert in DNA Gyrase B as well as a 45 aa conserved insert in SecA protein that were specific for the *Bacteroidetes *species [30]. In *Chlorobi *as well as *Chloroflexi *species, their light harvesting pigments are located in unique membrane-attached sac-like structures referred to as 'chlorosomes' [22,24,31,32]. A number of genes involved in the synthesis of chlorosomes components in *Chlorobi *have been identified by genomic and mutational analysis [26] and a few of them, viz. Fenna-Matthew-Olson (FMO) protein [33], are unique for this group [32,34]. However, the number of characteristics that are either unique to species from these two phyla, or are commonly shared by members of these phyla, are very limited. In the past few years, complete genomes of several *Bacteroidetes *and *Chlorobi *species have become available (see Table 1). Additionally, sequencing of genomes for many other *Bacteroidetes/Chlorobi *species is at different stages of completion (see Table 1), but considerable sequence information for these genomes is available in the NCBI database.

**Table 1:** General Characteristics of *Bacteroidetes/Chlorobi *Genomes

	**Strain Name**	**Taxonomic Order**	Genome Size (Mb)**	**GC Content (%)**	**Protein Number**	**Reference#**
**Bacteroidetes**	*Porphyromonas gingivalis *W83^#^	Bacteroidales	2.34	48.3	1909	[58]
	*Bacteroides fragilis *NCTC 9343^#^		5.24	44	4184	[16]
	*Bacteroides fragilis *YCH46^#^		5.31	33.5	4578	[35]
	*Bacteroides thetaiotaomicron *VPI-5482^#^		6.29	42	4778	[15]
	*Gramella forsetii *KT0803^#^	Flavobacteriales	3.8	36.6	3559	[59]
	Flavobacteria bacterium BBFL7*		5	35.0	2592	a
	*Flavobacteriales bacterium *HTCC2170*		5	37.0	3478	a
	*Flavobacterium johnsoniae *UW101*		-	35.2	4985	DOE-JGI
	*Flavobacterium sp*. MED217*		5	39.8	3735	a
	*Cellulophaga sp*. MED134*		5	38.2	2944	a
	*Croceibacter atlanticus *HTCC2559*		5	33.9	2719	a
	*Polaribacter irgensii *23-P*		-	31.0	2557	a
	*Psychroflexus torquis *ATCC 700755*		5	32–33.0	6751	a
	*Robiginitalea biformata *HTCC2501*		5	56.4	3228	a
	*Tenacibaculum sp*. MED152*		5	30.6	2679	a
	*Cytophaga hutchinsonii *ATCC 33406^#^	Sphingobacteriales	4.43	38.8	3785	CP000383
	*Salinibacter ruber *DSM 13855^#^		3.59	66.5	2801	[36]
**Chlorobi**	*Chlorobium chlorochromatii *CaD3^#^	Chlorobia	2.57	44.3	2002	CP000108
	*Chlorobium limicola *DSM 245*		2.4	51.3	2435	DOE-JGI
	Chlorobium phaeobacteroides BS1*		2.	45.5	3791	DOE-JGI
	*Chlorobium phaeobacteroides *DSM 266*		2.4	48.3	2789	DOE-JGI
	*Chlorobaculum tepidum *formerly *Chlorobium tepidum *TLS^#^		2.15	56	2252	[24]
	*Chlorobium luteolum *formerly *Pelodictyon luteolum *DSM 273^#^		2.36	57.3	2083	CP000096
	*Chlorobium clathratiforme *formerly *Pelodictyon phaeoclathratiforme *BU-1*		-	48.1	2762	DOE-JGI
	*Prosthecochloris aestuarii *DSM 271*		-	50.1	2313	DOE-JGI
	*Chlorobium phaeovibrioides *formerly *Prosthecochloris vibrioformis *DSM 265*		-	53.0	1747	DOE-JGI

^#^Indicates a completely sequenced genome. The references to the published genomes are provided. For others that are fully sequenced but not published, accession numbers for the genomes are given.

* Indicates that these genomes are at draft assembly stages. The information regarding genome sizes etc. in these cases have been obtained from the NCBI microbial sequence database.

The revised names for a number of *Chlorobi *species are as proposed in Ref. 45.

**# **The sequences marked 'a' are being sequenced by Gordon and Betty Moore Foundation Marine Biotechnology Intiative; DOE-JGE, indicates Department of Energy Joint Genome Institute. For some of the completed genomes, which are not published, their accession numbers are provided.

The availability of genomic sequences provide an opportunity to carry out in depth studies to identify novel molecular characteristics that are unique to these groups of bacteria and can be used for their diagnostics as well as biochemical and functional studies. Earlier comparative genomic studies on *Bacteroidetes/Chlorobi *species have been limited to only a few species and they have focused on specific aspects. The studies by Kuwahara [35] and Cerdeño-Tárraga et al. [16], who sequenced the genomes of *B. fragilis *strains, revealed that these genomes contained extensive DNA inversions in comparison to *B. thetaiotaomicron*. These inversion events are indicated to be important in terms of generating cell surface variability in these bacteria to avoid their recognition by the immune system. Large expansion of genes involved in the biosynthesis of cell surface polysaccharides and other antigens was also noted in these genomes [16,35]. A comparative analysis by Eisen et al. [24] of *C. tepidum *TLS genome identified many probable cases of lateral gene transfers (LGTs) between this species and *Archaea*; in all about 12% of *C. tepidum*'s proteins were indicated to be most similar to those from the archaea. Similarly, the analysis of *S. ruber *genome by Mongodin et al. [36] has identified many cases of potential LGT between *S. ruber *and haloarchaea, particularly involving the rhodopsin genes.

In our recent work, we have used comparative genomics to systematically identify various proteins that are uniquely found in either all members, or particular subgroups, of a number of important groups of prokaryotes. These studies have identified large number of proteins that are specific for alpha proteobacteria [37], chlamydiae [38], Actinobacteria [39], epsilon proteobacteria [40] and Archaea [41]. Such genes and proteins, because of their specificity for different phylogenetic or taxonomic groups, provide novel means for diagnostics and evolutionary studies [38,39,42-44] and for the discovery of important biochemical and physiological characteristics that are unique to these groups of prokaryotes. However, thus far no comparative study has examined different genes/proteins that are uniquely present in species from the *Bacteroidetes *and *Chlorobi *phyla or are commonly shared by species from these two groups. In order to identify proteins that are uniquely found in the *Bacteroidetes *and/or *Chlorobi *groups of species, we have carried out systematic blast searches on all open reading frames in the genomes of *P. gingivalis *W83, *B. fragilis *YCH46, *B. thetaiotaomicron *VPI-5482, *G. forsetii KT0803*, *C. luteolum *DSM 273 and *C. tepidum *TLS against all available sequences in the NCBI non-redundant (nr) database. This has led to identification of large numbers of proteins that are distinctive characteristics of species from different taxonomic groups within the *Bacteroidetes *phylum (e.g. specific for the *Bacteroides *genus, specific for the *Bacteroidales *and *Flavobacteriales *orders, or specific for the entire *Bacteroidetes *phylum). Additionally, large numbers of proteins that are specific for the *Chlorobi *species as well as some proteins that are uniquely shared by the *Bacteroidetes *and *Chlorobi *phyla have also been identified. This work also describes three conserved indels in important housekeeping proteins (viz. alanyl-tRNA synthetase, DNA polymerase subunit III and ClpB) that are distinctive characteristics of either the  *Chlorobi *or the* Bacteroidales-Flavobacteriales*-*Flexebacteraceae *groups. Phylogenetic analyses of the *Bacteroidetes *and *Chlorobi *were also carried out based on a concatenated sequence alignment for 12 highly conserved proteins and the results of these analyses support the inferences derived from the species distribution of various molecular signatures.

## Results

### Phylogenetic analyses of Bacteroidetes and Chlorobi species

Table 1 lists some characteristics of various *Bacteroidetes *and *Chlorobi *genomes that have been completely sequenced as well as for many others that are currently being sequenced. The taxonomy of the *Chlorobi *species has undergone significant revision in the past few years leading to name changes and new taxonomic groupings for a number of species [45]. The newly proposed and former names for some of the species that will be discussed in the present work are as follows: *Chlorobium tepidum *changed to *Chlorobaculum tepidum*; *Pelodictyon luteolum *changed to *Chlorobium luteolum*; *Prosthecochloris vibrioformis *changed to *Chlorobium phaeovibrioides*; *Pelodictyon phaeoclathratiforme *changed to *Chlorobium clathratiforme*. Although many of these species in the databases are still referred to by their former names, we have used the revised nomenclature in our work to interpret the results of evolutionary and comparative genomic studies.

Prior to undertaking comparative analyses of *Bacteroidetes *and *Chlorobi *genomes, phylogenetic analyses on these species were carried out to get an overview of their evolutionary relationships, which can serve as a reference point for comparative genomic analyses. Phylogenetic analysis of *Bacteroidetes *and *Chlorobi *species has been carried out previously using 16S rRNA sequences and a few isolated protein sequences [27-30,46-49]. However, recent studies show that analyses based on larger dataset derived from multiple genes/proteins sequences provide a more reliable phylogenetic inference [50]. Hence, phylogenetic analyses for these species was carried out based on concatenated sequences for 12 highly conserved proteins involved in a broad range of functions (see Methods section). The final sequence alignment for phylogenetic analysis contained a total of 6998 aligned positions.

**Figure 1:**
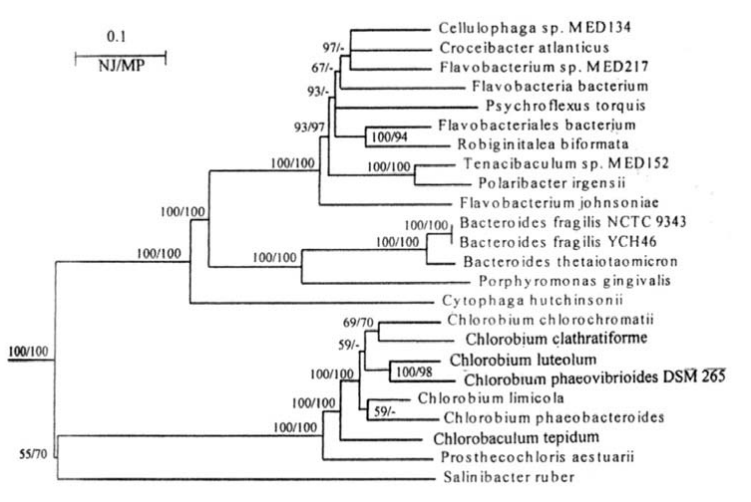
Neighbour-joining tree based on concatenated sequences for 12 highly conserved proteins. The tree was rooted using sequences for *Deinococcus-Thermus *species and numbers on the nodes indicate bootstrap scores in the NJ and maximum-likelihood analyses (NJ/MP). The branching position of *G. forsetii*, which became available after this analysis was completed, is not shown. However, our analysis of a smaller dataset indicates that it exhibits closest affinity for the flavobacteria *Psychrobacter torquis *(results not shown).

Phylogenetic trees were constructed using the neighbour-joining (NJ), maximum-likelihood (ML) and maximum-parsimony (MP) methods [51]. The results of these analyses for the NJ and ML methods are presented in Fig. 1. The trees were rooted using the sequences for *Deinococcus-Thermus *species. The tree in both cases consisted of two well-resolved clades (100% bootstrap scores by both treeing methods), one comprising of various *Chlorobi *species and the other containing various Cytophaga-Flavobacteria-Bacteroides (CFB) species. In these trees, *S. ruber *appeared as a deep branching outgroup of the *Chlorobi *clade, but in view of the low bootstrap score of the node indicating this relationship and the long branch that separated them, this relationship was not reliable. The topology of various species in the MP tree was very similar, except that in this tree *S. ruber *formed the outgroup of *Chlorobi *as well as various other CFB group of bacteria (results not shown). In addition to the uncertain position of *S. ruber*, different species belonging to the genus *Flavobacterium *did not form a coherent phylogenetic group (Fig. 1). For most other *Bacteroidetes *species, sequence information for multiple species from the same genera was not available.

Phylogenetic analysis on the concatenated dataset was also performed employing the character compatibility approach [52]. In this approach, all sites in the sequence alignment where only two amino acid states are found, with each state present in at least two species, are examined for mutual compatibility to find the largest clique of mutually compatible characters [52-56]. By removing all homoplasic as well as fast-evolving characters from dataset, this approach provides a powerful means for obtaining correct topology in difficult to resolve cases [56,57]. Our concatenated dataset for the 12 proteins contained 832 positions where only two amino acids were found, with each amino acid present in a minimum of two species. The mutual compatibility of these binary state sites was determined as described in the Methods section.

**Figure 2:**
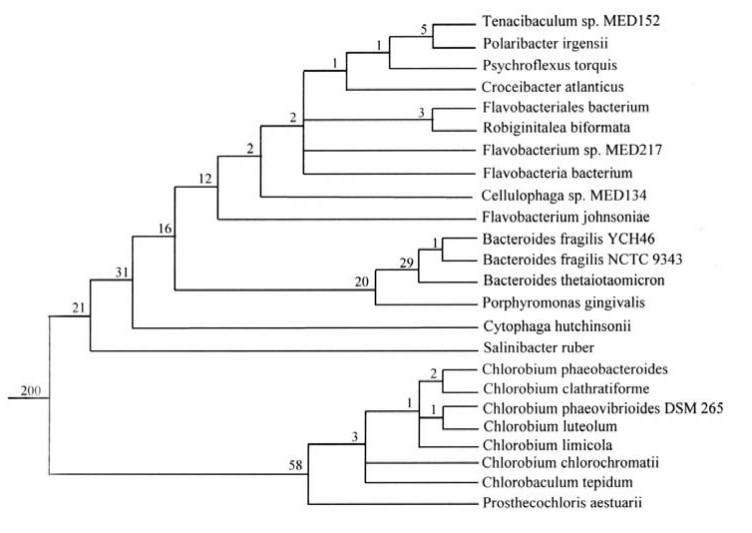
Character compatibility tree (or the largest clique of mutually compatible characters) based on two states sites in the concatenated sequence alignment for the 12 proteins. The clique consisted of 410 mutually compatible characters. The numbers of characters that distinguished different clades are indicated on the nodes. Rooting was done using the sequences for *Deinococcus-Thermus *species.

The compatibility analysis identified two largest sets of compatible characters (referred to as cliques) each containing 410 characters. These cliques were identical in all respects except that the relative branching positions of *Chlorobaculum tepidum *and *Chlorobium chlorochromatii*, which differed by a single character, were interchanged. A composite of these cliques, in which the branching positions of these two species are not resolved, is shown in Fig. 2. A large number of characters (i.e. 200) in this clique distinguished the *Chlorobi-Bacteroidetes *species from the two *Deinococcus-Thermus *species, which were included to serve as outgroup. The clique is comprised of two main clades, one consisting of various species belonging to the *Bacteroidetes *group and the other of different *Chlorobi *species. The species from each of these clades were distinguished by a large numbers of characters. In the *Bacteroidetes *clade, *S. ruber *was found to be the deepest branching lineage and its specific association with other *Bacteroidetes *species was supported by 21 uniquely shared characters, which is a highly significant result [57]. Additionally, the two main orders within the *Bacteroidetes *viz. *Bacteriodales *and *Flavobacteriales*, for which sequence information is available from multiple species, were clearly distinguished based upon multiple characters. However, these analyses detected no uniquely shared character between the *C. hutchinsonii *and *S. ruber*. Although, these two species are currently placed in the order *Sphingobacteriales*, phylogenetic trees do not support a specific grouping of them (see Fig. 1). Different *Flavobacterium *species again did not group together indicating that this genus does not constitute a phylogenetically coherent taxon. Within the *Chlorobi *clade, *Prosthecochloris aesturaii *was found to be the deepest branching lineage, but branching order of other *Chlorobi *species was not resolved.

### Comparative Genomic Studies on *Bacteroidetes *and *Chlorobi *Species

To identify proteins that are uniquely present in species from the *Bacteroidetes *and *Chlorobi *phyla at various taxonomic levels (genus and above), systematic Blastp searches were performed on each protein or ORF in the genomes of the following species: *Bacteroides *(*P. gingivalis *W83 [58], *B. fragilis *YCH46 [35], *B. thetaiotaomicron *VPI-5482 [15]); *Flavobacteria *(*G. forsetii str*. KT0803 [59]); *Chlorobi *(*C. luteolum *DSM 273 and *C. tepidum *TLS [24]). These analyses have identified a large number of proteins that are specific for different taxonomic groups. A brief description of these signature proteins and their evolutionary significances are discussed below.

#### Proteins (or ORFs) that are Specific for the Species within the Bacteroidetes Phylum

The blast searches on various ORFs from *P. gingivalis*, *B. fragilis *YCH46 and *B. thetaiotaomicron *genomes have identified 27 proteins that are present in most of the species from the *Bacteroidetes *phylum (Table 2). In addition to most fully sequenced *Bacteroidetes *genomes, the homologs of these proteins are also present in most *Bacteroidetes *species whose genomes have been partially sequenced. One of these proteins, PG0448, is present in all of the *Bacteroidetes *species, whose genomes have been either partially or fully sequenced. Two other proteins, PG1850 and PG2092, are present in all fully as well as partially sequenced genomes, except those from the *Bacteroides *genus. The absence of these proteins in only the species from this genus is very likely due to selective gene loss from this lineage and their genes also likely originated in a common ancestor of the *Bacteroidetes *phylum. Similarly, four proteins viz. PG0449, PG0779, PG1679 and PG2066, are present in virtually all other *Bacteroidetes *genomes, but they are missing in *C. hutchinsonii*. Their absence again is very likely due to selective gene loss from *C. hutchinsonii*. Eight additional proteins viz. PG0202, PG0362, PG0399, PG0482, PG0621, PG01281, PG1367 and PG1394, are present in all other fully as well as partially sequenced *Bacteroidetes *genomes, but they are only missing in *S. ruber*. The absence of these proteins in *S. ruber *can also be explained by selective gene loss. However, in view of the fact that *S. ruber *branches very deeply in comparison to all other *Bacteroidetes *species (Figs. 1 and 2), it is also possible that their genes evolved in a common ancestor of the other *Bacteroidetes *species after the divergence of *S. ruber*.

**Table 2:** Proteins that are Specific for the Phylum Bacteroidetes

**Protein Name**	**Accession No.**	*Length*	**Possible/Predicted Function**	*Comments*
PG0202	NP_904537	165	Uroporphyrinogen-III synthase HemD, putative; COG1587, HemD; pfam02602, HEM4	Missing in *S. ruber*^+^
PG0362	NP_904673	722	Hypothetical	Missing in *S. ruber*
PG0399	NP_904705	156	Putative lipoprotein	Missing in *S. ruber*
PG0448	NP_904748	434	Toluene × outer membrane/transport protein (OMPP1/FadL/TodX); pfam03349	All species present
PG0449	NP_904749	441	TPR domain protein; cd00189, TPR; COG3071, HemY; COG4783, Putative Zn-dependent protease	Not found in *F. johnsoniae *and *C. hutchinsonii*
PG0482	NP_904777	143	Hypothetical protein	Missing in *S. ruber*
PG0621	NP_904906	182	Hypothetical protein	Missing in *S. ruber*
PG0779	NP_905041	157	ExbD, Biopolymer transport protein; COG0848	Not found in *F. bacterium HTC*, *F. johnsoniae *and *C. hutchinsonii*
PG1281	NP_905462	387	Putative DNA mismatch repair protein; pfam01713, Smr; COG1193, Mismatch repair ATPase	Not found in *C. atlanticus *and *S. ruber*^+^
PG1367	NP_905532	200	Hypothetical protein	Missing in *S. ruber*
PG1394	NP_905555	165	Putative trans-membrane	Missing in *S. ruber*
PG1626	NP_905755	554	Putative hemin receptor	Missing in *C. hutchinsonii*; also present in *C. phaeobacteriodes*.
PG1679	NP_905797	464	Putative trans-membrane	Not found in *P. ruminicola *and *C. hutchinsonii*
PG1850	NP_905940	302	Hypothetical protein	Missing in *Bacteroides *species^+^
PG2066	NP_906128	351	Putative lipoprotein	Missing in *P. ruminicola *and *C. hutchinsonii*
PG2092	NP_906153	419	Hypothetical protein	Missing in *Bacteroides *species
BF0296	YP_097579	988	Outer membrane assembly protein	Missing in *P. gingivalis *and *S. ruber*^+^
BF0439	YP_097722	565	Putative outer membrane protein probably involved in nutrient binding	Missing in *P. gingivalis*, and *Tenacibaculum*
BF0534	YP_097817	192	Putative acetyl-transferase	Missing in *P. gingivalis, C. atlanticus *and *S. ruber*
BF0665	YP_097947	531	Putative exported protein	Missing in *P. gingivalis*, and *C. hutchinsonii*
BF0751	YP_098036	577	Putative exported protein	Missing in *P. gingivalis, P. torquis, R. biformata *and *C. hutchinsonii*
BF1057	YP_098341	506	Putative exported protein	Missing in *P. gingivalis*, and *C. hutchinsonii*
BF1254	YP_098538	507	Putative exported protein	Missing in *P. gingivalis*, and *C. hutchinsonii*
BF1327	YP_098610	514	Putative exported protein	Missing in *P. gingivalis*, and *C. hutchinsonii*
BF3185	YP_100464	490	Putative exported protein	Missing in *P. gingivalis*, and *C. hutchinsonii*
BF3612	YP_100889	542	Putative exported protein	Missing in *P. gingivalis *and *C. hutchinsonii*
BF4330	YP_101602	538	Putative exported protein	Missing in *P. gingivalis,, R. biformata *and *C. hutchinsonii*

For the proteins listed here, all significant blast hits are from various *Bacteroidetes *species, except as noted below. These proteins are present in all of the *Bacteroidetes *species listed in Table 1, except as noted in the comments.

^+^For the protein PG0202, a significant hit is also observed from *C. phaeobacteroides*; For BF0296 significant hits also observed from *P. aestuarii *and *C. phaeobacteroides*.

Of the proteins listed here, the following proteins are homologous to each other: BF0751, BF1057, BF1327, BF3185; BF1254, BF3612, BF4330.

We have also come identified a 3 aa deletion in a conserved region of ClpB protease that is present in all other *Bacteroidetes *species, except *S. ruber *(Additional file 1). Similar to the genes for the above 8 proteins, this deletion likely occurred in a common ancestor of the other *Bacteroidetes *species after the branching of *S. ruber*. Besides *Bacteroidetes *species, this indel is also present in the ClpB homologs from *C. phaebacteroidetes *(only *Chlorobi *species containing this protein) and the archaeum *Methanospirillum hungatei*, which is likely due to LGT. The remaining proteins in Table 2, of which 7 (BF0751, BF1057, BF1327, BF3185; BF1254, BF3612, BF4330) are homologous to each other, are missing in 1 or 2 sequenced species (e.g. *P. gingivalis, B. fragilis*, *C. hutchinsonii *or *S. ruber*) and their distribution pattern can also be explained by similar mechanisms as discussed above. Except for a few proteins that show limited similarity to conserved domains (CDs) found in other proteins [60], most of the proteins in Table 2 are of unknown function.

These searches have also identified several proteins that at present appear unique for the species from the *Bacteroidales *and *Flavobacteriales *orders. These proteins are listed in Table 3. Of these, the first 4 proteins viz. PG0336, PG1302, PG1537, PG2030 are present in nearly all complete as well as partially sequenced species from the above two orders, but they are not found in *S. ruber *as well as *C. hutchinsonii*. The latter two species, which show much deeper branching than all other *Bacteroidales *species (Figs. 1 and 2), are currently placed in the order *Sphingobacteriales*. The genes for the proteins listed in Table 3 have thus likely originated in a common ancestor of the *Bacteroidales *and *Flavobacteriales *after the divergence of *Sphingobacteria*. Thirty-seven additional proteins in Table 3 are also uniquely present in either all or many of the sequenced *Bacteroidales *species and a small number of flavobacteria species including *G. forsetii*. A large number of these proteins are only missing in *P. gingivalis*, which is likely due to gene loss. Of the proteins listed in Table 3, seven are indicated to be conjugative transposon proteins: TraJ, TraN, TraK, TraF, TraE and TraB (PG1251 and PG1479, PG1475, PG1478, PG1482, PG1483 and BF0127, respectively). Four of them are present in two clusters very close to each other (PG1478-PG1479, PG1482-PG1483), supporting their involvement in related functions [61,62]. The genes for these proteins have also likely evolved in a common ancestor of the *Bacteroidales *and *Flavobacteriales*, followed by gene losses in various species.

**Table 3:** Proteins that are Specific for the Bacteroidales and Flavobacteriales Orders

**Genome ID No. [Accession No.]**	**Possible/Predicted Function**	**Genome ID No. [Accession No.]**	**Possible/Predicted Function**
PG0336 [NP_904650]	Hypothetical protein	PG1276 [NP_905457]	DNA-binding protein, histone-like family
PG1302 [NP_905476]	Hypothetical protein	PG1389 [NP_905551]	DNA-binding protein, histone-like family
PG1537 [NP_905677]	Hypothetical protein	PG1444 [NP_905595]	Hypothetical protein
PG1251 [NP_905435]	Conjugative transposon protein TraJ	PG1475 [NP_905621]	Conjugative transposon protein TraN
PG2030 [NP_906097]	Hypothetical protein	PG1478 [NP_905624]	Conjugative transposon protein TraK
PG1492 [NP_905638]	Hypothetical protein	PG1479 [NP_905625]	Conjugative transposon protein TraJ
PG0302 [NP_904618]	Hypothetical protein	PG1482 [NP_905628]	Conjugative transposon protein TraF
PG0330 [NP_904645]	DNA-binding protein, histone-like family; smart00411, BHL	PG1483 [NP_905629]	Conjugative transposon protein TraE
PG0555 [NP_904845]	DNA-binding protein, histone-like family	PG1488 [NP_905634]	Hypothetical protein
PG0829 [NP_905084]	Hypothetical protein	PG1494 [NP_905640]	Hypothetical protein
PG0870 [NP_905118]*	Hypothetical protein	PG2040 [NP_906106]	DNA-binding protein, histone-like family
PG0875 [NP_905123]	TnpA; DNA replication, recombination & repair, COG2452	PG2127 [NP_906183]	Hypothetical protein
PG1206 [NP_905397]^c^	Mobilizable transposon, tnpC protein	BF1773 [YP_099054]*	Probable truncated integrase; cd01185, INT_Tn4399

**Missing in *Porphyromonas gingivalis *W83**			

BF0127 [YP_097410]	TraB	BF1727 [YP_099008]	Putative outer membrane protein maybe involved in nutrient binding
BF0136 [YP_097419]	Tetracycline resistance element mobilization: RteC	BF1860 [YP_099142]	Hypothetical protein
BF0146 [YP_097429]	Hypothetical protein	BF1926 [YP_211559]	Hypothetical protein
BF0319 [YP_097602]*	Putative exported protein	BF2130 [YP_099411]	Hypothetical protein
BF0342 [YP_097625]	Putative exported protein	BF2214 [YP_099495]	Hypothetical protein
BF1067 [YP_098351]	Hypothetical protein	BF3164 [YP_100443]	Putative lipoprotein
BF1422 [YP_098707]	Hypothetical protein	BF4258 [YP_101533]	Hypothetical protein
BF1567 [YP_098851]	Hypothetical protein		

The first five proteins in this table (viz. PG0336, PG1302, PG1537, PG1626, PG2030) are present in all *Bacteroidales *and *Flavobacteriales *species listed in Table 1; The other proteins are present in many of the *Bacteroidales *and *Flavobacteriales *species.

*Also present in *C. phaeobacteroides*.

The following proteins are homologous to each other: PG0330, PG0555, PG1276, PG1389, PG2040; PG0829, PG1444, PG1488; PG1251, PG1479; BF1422, BF1860; BF1926, BF4258.

#### Proteins that are specific for the order Bacteroidales or the genus Bacteroides

There are 4 genomes for the *Bacteroidales *species (*P. gingivalis *W83, *B. thetaiotaomicron *VPI-5482 and *B. fragilis *strains: NCTC 9343 and YCH46) that have been fully sequenced. Additionally, sequence information for a large number of genes/proteins from *Prevotella intermedia *17 and *Prevotella ruminicola *23, which belong to this order is available in the in NCBI database (see Table 1). Our blast searches on proteins from *P. gingivalis *genome have identified 52 proteins that are uniquely shared by either all or most of the sequenced *Bacteriodales *species and whose homologs are not found in any other species, except where noted (Table 4). Thirty-nine of these 52 proteins are uniquely found in all 4 fully sequenced *Bacteroidales *species. These species also form a strongly supported clade in phylogenetic trees (Figs. 1 and 2). Thus, it is likely that the genes for these proteins evolved in a common ancestor of this order. The remaining 13 proteins are lacking in at least one of the *Bacteroides *species (noted in Table 4), which is likely due to gene loss. In addition to the sequenced *Bacteroidales *species, high scoring homologs for many of the above proteins were also found in the two *Prevotella *species. These latter homologs were detected via genomic blasts against partially sequenced genomes from these species (see Methods).

**Table 4:** Proteins Unique to the Bacteroidales Order

**Genome ID No. [Accession No.]**	**Possible/Predicted Function**	**Genome ID No. [Accession No.]**	**Possible/Predicted Function**
PG0018 [NP_904375]^x^	hypothetical protein	PG1133 [NP_905341]	conserved hypothetical protein
PG0082 [NP_904431]^+^	putative exported protein	PG1139 [NP_905347]	conserved hypothetical protein; cd02966, Tlp_A_like_family
PG0125 [NP_904468]	conserved hypothetical protein	PG1214 [NP_905405]	hypothetical protein
PG0179 [NP_904515]^+^	putative exported protein	PG1301 [NP_905475]^+^	conserved hypothetical protein
PG0188 [NP_904523]^x^	lipoprotein, putative	PG1333 [NP_905502]^x^	putative exported protein
PG0216 [NP_904548]^+^	conserved hypothetical exported protein	PG1352 [NP_905517]	putative conserved hypothetical protein
PG0217 [NP_904549]^+^	cons. hypothetical exported protein	PG1388 [NP_905550]^+^	conserved hypothetical protein
PG0218 [NP_904550]^+^	conserved hypothetical exported protein	PG1441 [NP_905593]^x^	lysozyme-related protein; cd00737, endolysin_autolysin; COG3772, phage-related lysozyme
PG0246 [NP_904573]^+^	putative DNA-binding protein	PG1442 [NP_905594]	TraB
PG0312 [NP_904628]^+^	putative transmembrane protein	PG1458 [NP_905606]^x^	hypothetical protein
PG0326 [NP_904641]	hypoth; COG3637, Opacity protein & related surface antigens	PG1473 [NP_905619]^+^	conjugative transposon protein TraQ
PG0366 [NP_904677]^+#^	hypothetical protein	PG1621 [NP_905750]	conserved hypothetical exported protein
PG0434 [NP_904735]^+^	putative transmembrane protein	PG1757 [NP_905859]^x^	hypothetical protein
PG0541 [NP_904834]^+^	conserved hypothetical protein	PG1881 [NP_905968]^+^	putative lipoprotein
PG0574 [NP_904862]	hypothetical protein	PG1889 [NP_905974]^x^	hypothetical protein
PG0717 [NP_904988]^+^	lipoprotein, putative	PG1945 [NP_906027]^+^	conserved hypothetical protein
PG0781 [NP_905043]^+^	putative membrane protein	PG2006 [NP_906077]^+^	conserved hypothetical membrane protein
PG0816 [NP_905074]^x^	hypothetical protein	PG2079 [NP_906141]	conserved hypothetical protein
PG0831 [NP_905085]	Cons. protein maybe related to TraB	PG2083 [NP_906145]^+^	conserved hypothetical protein
PG0843 [NP_905095]^x^	conserved hypothetical protein	PG2116 [NP_906174]^x^	transposase
PG0851 [NP_905101]	Toprim domain protein	PG2130 [NP_906186]	FimX
PG0910 [NP_905150]	FHA domain protein, cd00060	PG2131 [NP_906187]^+^	60 kDa protein; OmpA, COG2885.2
PG0937 [NP_905172]	putative exported protein	PG2133 [NP_906189]^x^	lipoprotein, putative
PG0961 [NP_905192]^+^	conserved hypothetical protein	PG2149 [NP_906203]	putative conserved exported protein
PG1050 [NP_905267]^x^	putative lipoprotein	PG2168 [NP_906219]^+^	FimX
PG1125 [NP_905334]^+^	conserved hypothetical protein	PG2224 [NP_906265]^x^	hypothetical protein

All significant hits for these proteins are observed from the following *Bacteroidales *species, for which sequence information is available in the NCBI or TIGR microbial sequence database. *P. gingivalis *W83, *B. fragilis *NCTC 9343, *B. fragilis *YCH46 , *B. thetaiotaomicron *VPI-5482, *Prevotella intermedia *17, *P. ruminicola *23. Unless noted below, these proteins are present in all of the above-mentioned species. Of these proteins, the following are homologous to each other: PG0179, PG2133; PG0217, PG0218; PG0816, PG1458; PG0831, PG1442; PG2130, PG2168.

^+^Not found at present in either 1 or both *Prevotella *species.

^x^Missing in one of the *Bacteroides *species as well as *Prevotella *species.

^#^Also present was *C. phaeobacteroides *BS1.

The majority of the *Bacteroidales*-specific proteins are hypothetical and some of them are indicated as putative exported proteins (Table 4). Some interesting proteins in this list include the FimX proteins (PG2130, PG2168) that are involved in fimbriae production, which is necessary for adhesion to host surfaces [63]. Also of interest is the conserved hypothetical protein, PG1139, which shows slight but significant similarity to the conserved domain in the TlpA-like family, responsible for cytochrome maturation. One of the proteins PG0366 also had a significant hit from *C. phaeobacteroides*, which could be due to LGT [64]. There are seven proteins in Table 4 (PG0216-PG0218, PG1441-PG1442, PG2130-PG2131) that are present in clusters of two or three, suggesting that they could form functional units. Further, a number of proteins in this table (viz. PG0179, PG2133; PG0217, PG0218; PG0816, PG1458; PG0831, PG1442; PG2130, PG2168) are homologous to each other, indicating that they resulted from gene duplication events.

The *Bacteroides *genus contains three fully sequenced genomes corresponding to *B. thetaiotaomicron *VPI-5482 and *B. fragilis *strains NCTC 9343 and YCH46. The blast searches on *B. fragilis *YCH46 genome have identified 185 proteins that are mainly specific for these species (Additional file 2) and their homologs are not found in *P. gingivalis*. For 10 of these proteins, significant similarity was also observed for at least one of the two *Prevotella *species, suggesting that within the order *Bacteriodales*, species from the *Bacteroides *and *Prevotella *genera may be more closely related to each other in comparison to *P. gingivalis*. Most of these proteins are of unknown functions, however, some have been annotated as transmembrane or lipoproteins. Thirty-nine of these proteins are present in clusters of two to four in the genome indicating that they could be involved in related functions. A number of proteins in this table are homologous to each other indicating that they have likely resulted from gene duplication events.

#### Proteins that are specific for the order Flavobacteriales

The complete genome of the first flavobacteria species viz.*G. forsetii KT0803 *became available very recently [59]. However, sequence information for a large number of other *Flavobacteriales *species, whose genomes are being sequenced (see Table 1), is available in the NCBI database. Our blastp and PSI-blast searches on different ORFs in the *G. forsetii *genome have identified 38 proteins that are uniquely present in virtually all of the *Flavobacteriales *species (Table 5). Twenty-six of these proteins are present in all *Flavobacteriales *species listed in Table 1, whereas the remaining 12 are missing in only one of the species. An additional group of 146 proteins are also specific for the *Flavobacteriales*, but they are missing in some flavobacteria species or limited to only a small numbers of flavobacteria (Additional file 3). Because the genomes for most of the *Flavobacteriales *species are not complete at the present time, these proteins were not separated into different groups.

**Table 5:** Proteins that are Specific for Species from the Flavobacteriales Order

**orf No. [Accession No.]**	**Possible/Predicted Function**	**Genome ID No. [Accession No.]**	**Possible/Predicted Function**
orf89 [CAL65078]^b^	membrane protein	orf1826 [CAL66812]	hypothetical protein
orf92 [CAL65081]	secreted protein	orf1872 [CAL66858]	hypothetical protein
orf107 [CAL65096]	membrane protein	orf2280 [CAL67264]^c^	hypothetical protein
orf110 [CAL65099]	conserved hypothetical protein	orf2667 [CAL67651]	conserved hypothetical protein
orf191 [CAL65180]	membrane or secreted protein	orf2698 [CAL67682]	secreted protein
orf403 [CAL65392]^b^	hypothetical protein	orf2700 [CAL67684]	hypothetical protein
orf509 [CAL65498]^c^	hypothetical protein	orf2718 [CAL67702]	hypothetical protein
orf612 [CAL65599]^b^	secreted protein	orf2731 [CAL67715]^b^	secreted protein
orf983 [CAL65970]^a^	hypothetical protein	orf2756 [CAL67740]	secreted protein
orf995 [CAL65982]	phospholipid/glycerol acyltransferase; smart00563, PlsC	orf2825 [CAL67809]	secreted protein
orf998 [CAL65985]	membrane protein	orf2844 [CAL67828]	membrane protein
orf1059 [CAL66046]	membrane protein	orf2917 [CAL67899]	secreted protein
orf1078 [CAL66065]^a^	membrane or secreted protein	orf2939 [CAL67921]	hypothetical; COG1577, ERG12
orf1453 [CAL66440]	secreted protein	orf3043 [CAL68025]	hypothetical protein
orf1469 [CAL66456]^a^	secreted protein	orf3076 [CAL68058]^e^	hypothetical protein
orf1555 [CAL66542]	secreted protein	orf3240 [CAL68223]	membrane protein
orf1618 [CAL66605]^d^	secreted protein	orf3266 [CAL68249]^a^	conserved hypothetical protein
orf1766 [CAL66752]	hypothetical protein	orf3313 [CAL68296]	hypothetical protein
orf1776 [CAL66762]	hypothetical protein	orf3501 [CAL68484]	conserved hypothetical protein

Unless otherwise indicated, all significant hits for the proteins listed in this table are from the following *Flavobacteriales *species, for which sequence information is presently available. *G. forestii KT0803, F. bacterium BBFL7, F. bacterium HTCC2170, F. johnsoniae UW101, Flavobacterium sp. MED217, Cellulophaga sp. MED134, C. atlanticus HTCC2559, P. irgensii 23-P, P. torquis ATCC 700755, R. biformata HTCC2501, Tenacibaculum sp. MED152*. The proteins are present in all of these species except as noted below:

^a^Missing in *Polaribacter irgensii *23-P

^b^Missing in *Flavobacterium johnsoniae *UW101

^c^Missing in *Flavobacteria bacterium *BBFL7

^d^Missing in *Psychroflexus torquis *ATCC 700755

^e^Missing in *Robiginitalea biformata *HTCC2501

#### Proteins that are unique for the Chlorobi Phylum

The genomes of three *Chlorobi *species viz. *C. luteolum *DSM 273,*C. tepidum *TLS, and *C. chlorochromatii *CaD3, have been fully sequenced. Our blast analyses on the *C. tepidum *and *C. luteolum *genomes have identified 51 proteins that are uniquely shared by species from this phylum (Table 6). In addition to the 3 completely sequenced genomes, homologs of these proteins are also present in six others *Chlorobi *species (see Table 1), for which sequence information is available in the NCBI database. The genes for these proteins likely originated in a common ancestor of various *Chlorobi *species, which form a distinct, strongly supported, clade in phylogenetic trees (see Figures 1 and 2). The vast majority of these proteins are of hypothetical or unknown functions. However, 5 of them are indicated to be involved in functions related to photosynthesis. Of these, Plut_0264 and Plut_0265 are clustered in the genome and they correspond to chlorosome envelope proteins C and A, respectively. The protein Plut_1500, which is indicated as bacteriochlorophyll A protein, corresponds to the FMO protein that is involved in the attachment of chlorosomes to the cytoplasmic membrane [34]. The other two photosynthesis-related proteins, Plut_0620 and Plut_1628, are annotated as photosystem P840 reaction centre protein PscD and the photosystem P840 reaction centre cytochrome c-551, respectively [24,32]. Three additional chlorobi-specific proteins, Plut_1714-Plut_1716, are clustered together in the genome indicating that they may form a functional unit [61]. There are 65 additional proteins that are also specific for the *Chlorobi *species (Additional file 4). However, unlike the proteins in Table 6, these proteins are missing in a number of the *Chlorobi *species and their species distribution does not show any clear pattern and these could involve gene loss or LGTs [65]. However, the first 8 proteins listed in this additional file (viz. Plut_0107, Plut_0759, Plut_0762, Plut_0981, Plut_0985, Plut_1092, Plut_1145, Plut_1858) are only found in *C. luteolum *and *C. phaeovibrioides*. These two species form a strongly supported clade in various phylogenetic trees (Figs. 1 and 2) and a specific relationship between them is further supported by the unique presence of these shared proteins. For one of the proteins in this table, Plut_1345, a significant hit is also observed from *C. hutchinsonii*, which could be due to LGT [64,65].

**Table 6:** Proteins that are Specific for the *Chlorobi *Species

**Genome ID No. [Accession No.]**	**Possible/Predicted Function**	**Genome ID No. [Accession No.]**	**Possible/Predicted Function**
Plut_0059 [YP_373992]	Hypothetical protein	Plut_1225 [YP_375130]	Hypothetical protein
Plut_0074 [YP_374007]	orfCR	Plut_1238 [YP_375143]	Hypothetical protein
Plut_0111 [YP_374044]	Hypothetical protein	Plut_1332 [YP_375234]	TPR repeat
Plut_0145 [YP_374078]	Hypothetical protein	Plut_1409 [YP_375311]	Hypothetical protein
Plut_0160 [YP_374093]	Hypothetical protein	Plut_1465 [YP_375367]	Hypothetical protein
Plut_0264 [YP_374195]	chlorosome envelope protein C	Plut_1469 [YP_375371]	Hypothetical protein
Plut_0265 [YP_374196]	chlorosome envelope protein A; Bac_chlorC, pfam02043	Plut_1491 [YP_375393]	Hypothetical protein
Plut_0278 [YP_374209]	Hypothetical protein	Plut_1500 [YP_375402]	FMO, BchlA protein
Plut_0282 [YP_374213]	Hypothetical protein	Plut_1517 [YP_375417]	Hypothetical protein
Plut_0295 [YP_374226]	Hypothetical protein	Plut_1608 [YP_375505]	Srm
Plut_0325 [YP_374256]	Hypothetical protein	Plut_1625 [YP_375522]	Hypothetical protein
Plut_0409 [YP_374340]	Hypothetical protein	Plut_1628 [YP_375525]	Photosystem P840 reaction center cytochrome c-551
Plut_0422 [YP_374353]	Hypothetical protein	Plut_1682 [YP_375579]	Hypothetical protein
Plut_0489 [YP_374420]	Hypothetical protein	Plut_1714 [YP_375611]	Hypothetical protein
Plut_0499 [YP_374430]	Hypothetical protein	Plut_1715 [YP_375612]	Hypothetical protein
Plut_0540 [YP_374467]	Hypothetical protein	Plut_1716 [YP_375613]	Hypothetical protein
Plut_0572 [YP_374498]	Hypothetical protein	Plut_1725 [YP_375622]	Hypothetical protein
Plut_0620 [YP_374546]	Photosystem P840 reaction center protein PscD	Plut_1742 [YP_375639]	Hypothetical protein
Plut_0666 [YP_374587]	Hypothetical protein	Plut_1743 [YP_375640]	Hypothetical protein
Plut_0713 [YP_374634]	Hypothetical protein	Plut_1746 [YP_375643]	Hypothetical protein
Plut_0779 [YP_374695]	Hypothetical protein	Plut_1933 [YP_375818]	Hypothetical protein
Plut_0950 [YP_374855]	Hypothetical protein	Plut_2003 [YP_375888]	orfCR
Plut_1012 [YP_374917]	Hypothetical protein	Plut_2041 [YP_375926]	Hypothetical protein
Plut_1195 [YP_375100]	Hypothetical protein	Plut_2100 [YP_375985]	Hypothetical protein
Plut_1217 [YP_375122]	Hypothetical protein	Plut_2117 [YP_376002]	Hypothetical protein
Plut_1223 [YP_375128]	Hypothetical protein		

All significant Blast hits for these proteins are observed only from the following *Chlorobi *species for which sequence information is presently available: *C. luteolum *DSM 273, *C. tepidum *TLS, *C. chlorochromatii *CaD3, *C. limicola *DSM 245, *C. phaeobacteroides *BS1, *C. phaeobacteroides *DSM 266, *C. clathratiforme *BU-1, *P. aestuarii *DSM 271 and *C. phaeovibrioides *DSM 265. Of these proteins, the following proteins are homologous to each other:Plut_0059, Plut_1491; Plut_0074, Plut_2003.

**Figure 3:**
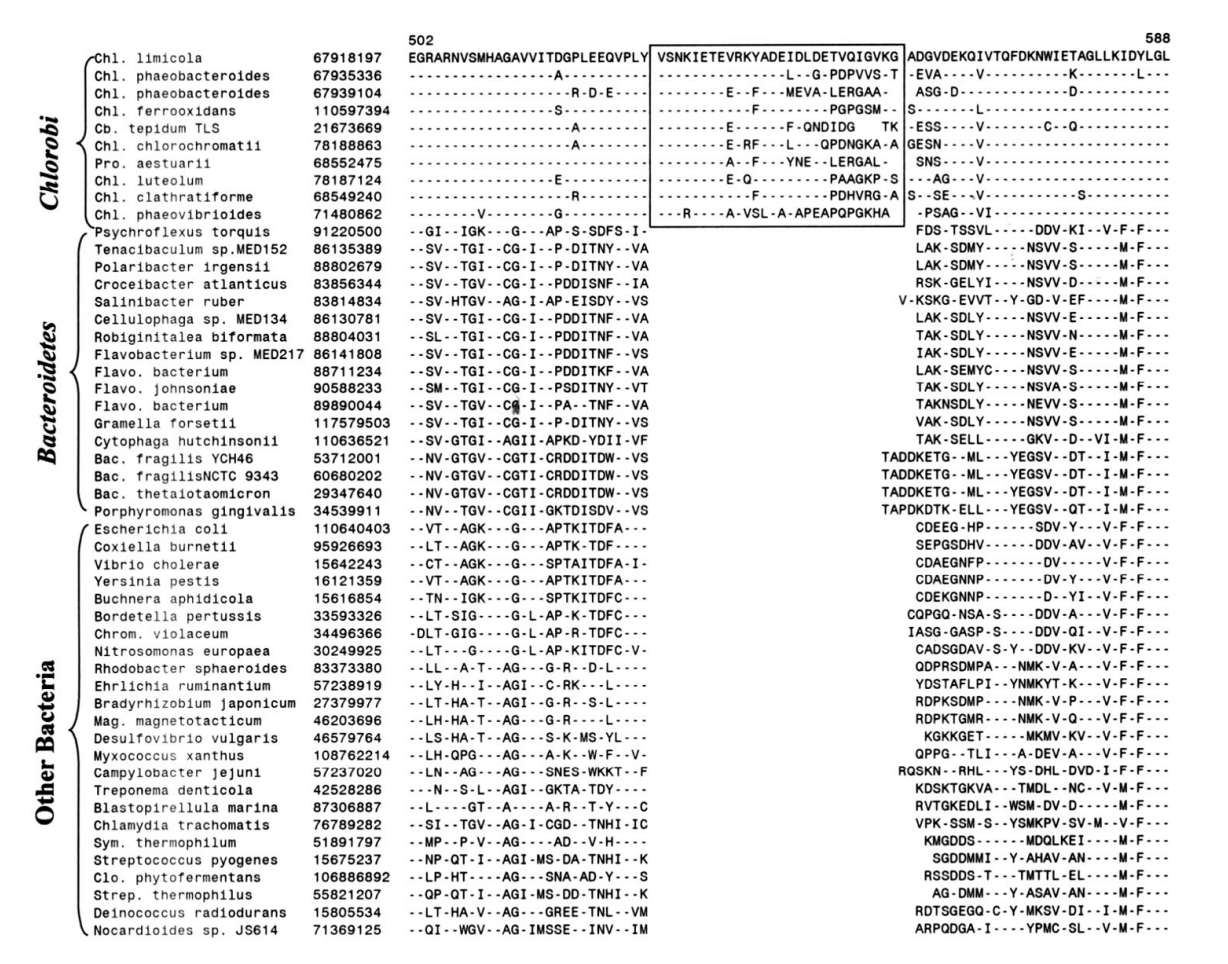
Partial sequence alignments of the DnaE protein showing a large insert of about 28 aa that is uniquely present in *Chlorobi *homologs. The dashes (-) denote identity with the amino acid on the top line. Except for the *Chlorobi *species, this insert is not found in any other organism. Sequence information for only representative species from other groups of bacteria is shown. Abbreviations in the species names are: *Bac., Bacteroides; Cb., Chlorobaculum; Chl., Chlorobium; Chrom., Chromobacterium; Clo., Clostridium; Pro., Prosthecochloris; Sym., Symbiobacterium; Flavo., Flavobacterium; Strep., Streptococcus*.

**Figure 4:**
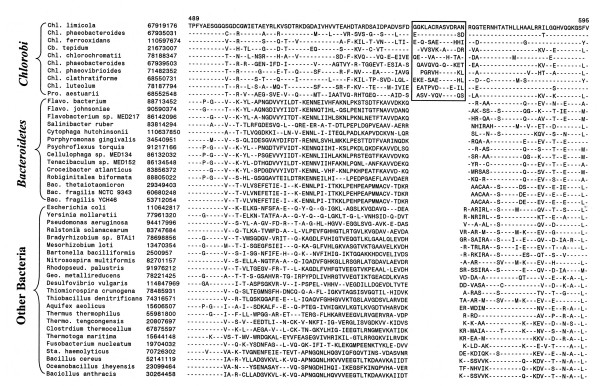
Partial sequence alignments of alanyl-tRNA synthetase showing a conserved insert of about 12–14 aa that is a distinctive characteristic of *Chlorobi *homologs and not found in other bacteria. The dashes (-) denote identity with the amino acid on the top line. Additional abbreviations: *Geo., Geobacter; Sta., Staphylococcus; Thermo., Thermoanaerobacter*.

In addition to the proteins that are uniquely found in various *Chlorobi *species, we have also identified two large conserved inserts in two widely distributed proteins that are distinctive characteristics of the *Chlorobi *phylum. The first of these signatures is a 28 aa insert in the DNA polymerase III alpha subunit encoded by the *dnaE *gene that is required for chromosomal replication in bacteria [66]. The large insert in DnaE is present in all of the *Chlorobi *homologs but it is not found in any other species (Fig. 3). A smaller insert of 3–4 aa is probably also present in this regions in some *Bacteroidales *species, but based on their different sizes and sequence characteristics, these inserts are of independent origin. The other *Chlorobi*-specific signature consists of a 12–14 aa insert in alanyl-tRNA synthetase (Fig. 4), which plays an essential role in protein synthesis. This insert is again present in all *Chlorobi *homologs but not found in any other species indicating that it provides a reliable molecular marker for this group.

#### Proteins that are uniquely shared by the Bacteroidetes and Chlorobi species

The *Bacteroidetes *and *Chlorobi *species generally branch very close to each other in phylogenetic trees [27-30]. However, there are very few characteristics known that are uniquely shared by species from these two phyla. Our analysis has identified 3 proteins (PG0081, PG0649 and PG2432 in Table 7) that are uniquely found in virtually all of the fully as well as partially sequenced *Bacteroidetes *and *Chlorobi *genomes. These results are significant because *Bacteroidetes *or *Chlorobi *species do not share any protein in common with different species from any other group of bacteria. Of these proteins, the protein PG0081 is also found in *Fibrobacter succinogenes*. A close and specific relationship of *F. succinogenes *to the *Bacteroidetes *and *Chlorobi *groups was strongly suggested by our earlier work based on conserved indels in different proteins [30]. This inference is reinforced by the unique presence of this protein in these different groups. The absence of the protein PG0649, which is present in all other *Bacteroidetes *and *Chlorobi *species, in *S. ruber*, is probably due to gene loss. Three other proteins, PG1818, PG1977 and BF2465 although they appear unique to the *Bacteriodales *and *Chlorobi*, their homologs are not detected in most *Flavobacteria *including *G. forsetii*. It is likely that the genes for these proteins also evolved in a common ancestor of the *Bacteroidetes *and *Chlorobi *phyla followed by gene losses in particular *Bacteroidetes *lineages. All of these proteins are of unknown functions except PG1818, which is annotated as a putative transmembrane protein with significant similarity to the conserved domain of the ResB-like family.

**Table 7:** Proteins that are Uniquely Shared by the *Bacteroidetes *and *Chlorobi *Species

**Protein I.D. No. [Accession No.]**	PG0081 [NP_904430]	PG0649 [NP_904929]	BF2432 [YP_099715]	PG1818 [NP_905917]	PG1977 [NP_906051]	BF2465 [YP_099748]
**Length (aa)**	725 aa	194 aa	1478	238 aa	668 aa	93 aa
**Possible Function**	Hypoth.	Hypoth.	Hypoth.	Putative transmembrane	Hypoth.	Hypoth.
***Bacteroidetes ***						
*P. gingivalis*	*	*	*	*	*	*
*B. fragilis *NCT	*	*	*	*	*	*
*B. fragilis*YCH	*	*	*	*	*	*
*B. thetaiotaomi*.	*	*	*	*	*	*
*Prev. intermedia*	*	*	*	*	*	*
*Prev. ruminicola*	*	*	*		*	*
*G. forestii*	*	*	*			
*F. bacterium *BBF	*	*	*			*
*F. bacterium *HTC	*	*	*			
*F. johnsoniae*	*	*	*			
Flavobacterium	*	*	*			
Cellulophaga	*	*	*			
*C. atlanticus*	*	*	*			
*Polibacter irgensii*	*	*	*			
*Psychro. torquis*	*	*	*			
*Rob. biformata*	*	*				
Tenacibaculum	*	*	*			
*Cyto. hutchinsonii*	*	*	*		*	*
*Salinibacter ruber*	*		*		*	
***Chlorobi***						
*Cb. tepidum*	*	*		*	*	
*C. chlorochrom*.	*	*	*	*	*	*
*C. luteolum*	*	*	*	*	*	*
*C. limicola*	*	*	*	*	*	
*C. phaeobac *BS1	*	*	*	*	*	
*C. phaeobac *DSM	*	*	*	*	*	
*C. clathratiforme*	*	*	*	*	*	*
*Prosthec. aesturii*	*	*	*	*	*	*
*C. phaeovibrioides*	*	*	*	*	*	

All significant blast hits for these proteins are from the indicated (marked by *) Bacteroidetes and Chlorobi species. For the protein PG0081, a homolog is also present in *Fibrobacter succinogenes subsp. succinogenes S85 *(identified by blast search against the partial sequence). The blank space indicates that no hit showing significant similarity was detected at the present time.

## Discussion

This work has identified a large number of proteins that are specific for *Bacteroidetes *and *Chlorobi *species at various taxonomic levels. Homologs exhibiting significant similarity to these proteins are not found in any other bacteria, except in a few isolated cases. Among the proteins that are specific for the *Bacteroidetes*, 27 proteins are specific for the entire phylum as their homologs are present in species from all three main orders within this phylum. Many other proteins are limited to various clades within the *Bacteroidetes *phylum. These include 41 proteins that are common to the *Flavobacteriales *and *Bacteroidales *orders; 53 and 38 proteins that are specific for the *Bacteriodales *and *Flavobacteriales *orders, respectively; and 185 proteins that are specific for the *Bacteriodes *genus. We have also identified 51 proteins that are specific for the *Chlorobi *species and 6 proteins that are uniquely shared by the *Bacteroidetes *and *Chlorobi phyla*. Two large conserved inserts in the DnaE and AlaRS proteins that are distinctive characteristics of the *Chlorobi *species were also discovered in this work. In addition, a deletion in ClpB protein that is mainly specific for the *Bacteriodales*, *Flavobacteriales *and *Flexibacteraceae *was also identified. In earlier work, a number of conserved inserts that are specific for either the *Bacteroidetes *phylum (viz. SecA and Gyrase B) or commonly shared by the *Bacteroidetes *and *Chlorobi *species were also described. Based upon their specificity for the *Bacteroidetes *and *Chlorobi *species, these molecular markers provide novel and more definitive means for identifying and circumscribing species from these groups.

**Figure 5:**
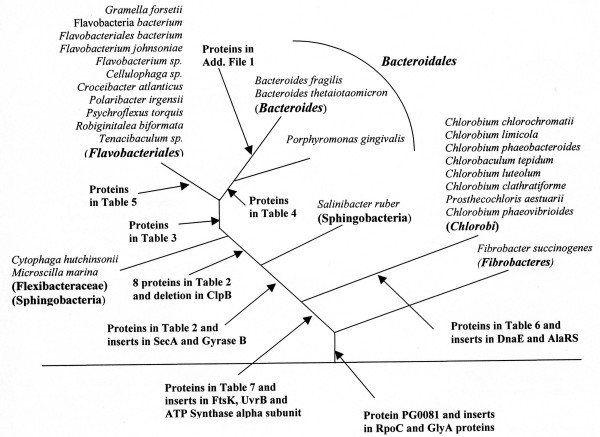
A summary diagram showing the evolutionary stages where different signature proteins and conserved indels that are specific for the *Bacteroidetes *and *Chlorobi *species have likely evolved or originated. Some of the conserved inserts that are specific for these groups or indicate their branching position relative to other bacterial phyla have been described in earlier work [30,43,69].

The species distribution patterns of these signature proteins and conserved indels strongly suggest that they or the genes for them have evolved at various stages in the evolution of these bacteria (Fig. 5). However, subsequent to their evolution or introduction in these genes, these genomic characteristics are stably retained in various descendents of these lineages with minimal gene loss or LGTs, as has also been found in other related studies [37-41,44,67,68]. The evolutionary relationship among the *Bacteroidetes *species as deduced from these signature proteins is in complete agreement with their branching pattern in phylogenetic trees (Figs. 1 and 2). The unique presence of several signature proteins as well as conserved indels in a number of essential proteins (viz. FtsK, UvrB and ATP synthase alpha subunit) by different *Bacteroidetes *and *Chlorobi *species provides compelling evidence that species from these two groups shared a common ancestor exclusive of all other bacteria. In earlier studies, a close relationship of *Fiborbacteres *to the *Bacteroidetes *and *Chlorobi *was also observed [2,30]. The species from all these three groups were found to contain large conserved indels in RNA polymerase β ' subunit and serine hydroxymethyl transferase, that were not found in any other bacteria [30]. The species from these three groups also branched in the same position based on distribution profiles of signature sequences in a number of other proteins and in different phylogenetic trees [30,69]. The unique shared presence of the protein PG0081 by all sequenced *Chlorobi *and *Bacteroidetes *species as well as *Fibrobacter succinogenes*, provides further evidence that species from these three groups form a single superphylum and that they shared a common ancestor exclusive of all other bacteria [30].

This paper also reports phylogenetic analyses of *Bacteroidetes *and *Chlorobi *species based on a concatenated alignment of 12 highly conserved proteins. The branching order of various species in the trees obtained using different phylogenetic methods were in general very similar with the clades corresponding to the *Chlorobi *species and the *Cytophaga-Flavobacteria-Bacteroides *species, well resolved from each other with 100% bootstrap scores. The species corresponding to the two main groups within the *Bacteroidetes *phylum (viz. the *Bacteriodales *and *Flavobacteriales *orders) were also clearly resolved. However, in the trees constructed by traditional phylogenetic methods such as NJ, ML and MP, the phylogenetic placement of *S. ruber *was not resolved. In all of these trees, *S. ruber *appeared either as a very deep branch of the *Chlorobi *clade (i.e. in NJ and ML trees) or as outgroup of both the *Chlorobi *and the CFB clades (MP tree). In contrast to these trees, when the same dataset was analyzed by means of the character compatibility or clique approach, *S. ruber *formed the deepest branch of the *Bacteroidetes *species and its specific association with this group was supported by 21 uniquely shared characters, indicating strongly that this affiliation was reliable [57]. These results provide evidence that the character compatibility approach, which removes all fast-evolving as well as homoplasic sites from a given dataset, provides a powerful means for obtaining correct topology in cases, such as that for *S. ruber*, whose phyletic affinity has proven difficult to establish by traditional phylogenetic methods [13,52,56,57].

The cellular functions of most of the *Bacteroidetes *or *Chlorobi*-specific proteins identified in the present work are not known. A few of the *Chlorobi-*specific proteins are involved in chlorosome- or photosynthesis-related functions, which is expected as *Chlorobi *is one of the few bacteria phyla that possesses photosynthetic ability [22,31,32,34,70]. A number of other proteins exhibit weak sequence similarity to conserved domains in certain other proteins, but considering that the overall sequence similarity is not significant, the actual functions of these proteins could be quite different. Therefore, an important task for the future is to determine the cellular functions of these *Bacteroidetes *or *Chlorobi *specific proteins. Likewise, it is also of much interest to determine the functional significance of the conserved indels in SecA, Gyrase B and ClpB proteins that are distinctive characteristics of the *Bacteroidetes *[30], and of the inserts in DnaE and AlaRS proteins that are specific for the *Chlorobi *species. The retention of these signature proteins and conserved indels by all species from these groups strongly suggests that they are functionally important for these bacteria. Hence, further studies on these molecular signatures should lead to the discovery of novel biochemical and physiological characteristics that are unique to these bacteria. The primary sequences of many of these genes/proteins that are specific for the *Bacteroidetes *or *Chlorobi *species are highly conserved and they provide novel means for identification of both known as well as novel species belonging to these groups by means of PCR-based and immunological methods. Several *Bacteroidetes *species play central role in the initiation and progression of periodontal diseases in humans [12,18,19,58]. Hence, the proteins that are specific to these bacteria also provide important potential targets for development of therapeutics and vaccines for treatment and prevention of periodontal diseases.

## Methods

### Identification of Proteins that are Specific for Bacteroidetes and Chlorobi

The blastp searches were carried out on each ORF in the genomes of *P. gingivalis *W83 [58], *B. fragilis *YCH46 [35], *B. thetaiotaomicron *VPI-5482 [15], *G. forsetii KT080 *[59], *Chlorobium *(*Pelodictyon*)*luteolum *DSM 273 and *C. tepidum *TLS [24], to identify proteins that are specific for the *Bacteroidetes *and *Chlorobi *phyla at different taxonomic levels. The blastp searches were performed against all organisms (i.e. using the NCBI non-redundant (nr) database) with default settings except that the low complexity filter was not used [71]. The proteins that were of interest were those where either all significant hits were from these groups of species or which involved a large increase in E values from the last *Bacteroidetes-Chlorobi *hit to the first hit from any other organism and the E values for the latter hits in most cases > 10^-4^, which indicates a weak similarity that could occur by chance. However, higher E values were sometimes acceptable particularly for smaller proteins as the magnitude of the E value depends upon the length of the query sequence. All promising proteins were further analyzed using the position-specific iterated (PSI)-blast program [71]. This program creates a position-specific scoring matrix from statistically significant alignments produced by the blastp program and then searches the database using this matrix. The PSI-blast is more sensitive in identifying weak but biologically relevant sequence similarity as compared to the blastp program [71]. In the present work, a protein was considered to be specific for a given group if all hits producing significant alignments were from that group of species. However, we have also retained a few proteins where 1 or 2 isolated species from other groups of bacteria also had acceptable E values, as they provide possible cases of lateral gene transfer. For all of the *Bacteroidetes *or *Chlorobi*-specific proteins identified in the present work, their protein ID's, accession numbers and any information regarding cellular functions (such as COG number or the presence of any conserved domain) are presented here. Preliminary sequence information regarding the presence of a homolog of a query protein in the partially sequenced genomes of *P. intermedia, P. ruminicola *and *F. succinogenes subsp. succinogenes S85 *were obtained via genomic blasts against The Institute for Genomic Research database for unfinished microbial genomes [72]. In describing various proteins in the text, "PG," "BF," "BT," "orf", "Plut" and "CT" indicate the identification numbers of the proteins in the genomes of *P. gingivalis *W83, *B. fragilis *YCH46, *B. thetaiotaomicron *VPI-5482,*G. forsetii KT080, C. (Pelodictyon) luteolum *DSM and *C. tepidum *TLS, respectively.

### Phylogenetic Analysis

The amino acid sequences for the 12 conserved proteins viz. RNA polymerase β subunit, RNA polymerase β ' subunit (RpoC), alanyl-tRNA synthetase (AlaRS), arginyl-tRNA synthetase, phenylalanyl-tRNA synthetase, elongation factor-Tu, elongation factor G, RecA protein, DNA gyrase subunit A, DNA gyrase subunit B, Hsp60 or GroEL protein and DnaK or Hsp70 protein, for different species were downloaded from the NCBI database and aligned using the ClustalX (1.83) program using the default settings [73]. The sequences for two deep-branching species, *D. radiodurans *and *T. aquaticus *[27], were included in this dataset for rooting purposes. The sequence alignments for all 12 proteins were concatenated into a single large alignment containing 8899 positions. Poorly aligned regions from this alignment were removed with the Gblocks 0.91b program [74], using the default settings, except that allowable gap position was selected to half. This resulted in a final sequence alignment of 6998 sites, which was used for phylogenetic analyses. A NJ tree based on this alignment (bootstrapped 1000 time) was constructed based on Kimura's model [75] using the TREECON programs [76]. Maximum-likelihood and MP trees were computed using the WAG+F model plus a gamma distribution with four categories [77] using the TREE-PUZZLE [78] and Mega 3.1 program [79], respectively. All trees were bootstrapped 100 times [80], unless otherwise indicated.

The character compatibility analysis on the concatenated alignment was carried out as described recently [57]. Using the program "DUALSITE" [57], all sites in the alignments where only two amino acid states were found, with each state present in at least two species, were selected. All columns with any gaps were omitted. The sites where one of the states is present in only a single species are not useful for compatibility analysis. All useful two state sites were converted into a binary file of "0, 1" characters using the DUALSITE program and this file was used for compatibility analysis [57]. The compatibility analysis was carried out using the CLIQUE program from the PHYLIP (ver. 3.5c) program package [81] to identify the largest clique(s) of compatible characters. The cliques were drawn and the numbers of characters that distinguished different nodes were indicated. The sequence information for *G. forsetii*, which became available after these analyses were completed [59] is not included in these trees.

### Identification of conserved indels

Multiple sequence alignments for large numbers of proteins have been created in our earlier work [82-84]. These alignments were visually inspected to search for any indels in a conserved region that was uniquely present in *C. tepidum *(the only *Chlorobi *species present in these groups). The specificity of any potential indel for these groups was evaluated by carrying out by blastp searches on a short segment of the sequence (between 80–120 aa) containing the indel and the flanking conserved regions against the nr database. The purpose of these blast searches was to obtain information from all available homologs to determine the specificities of the indels.

## Abbreviations

CD, conserved domain; CFB, Cytophaga-Flavobacteria-Bacteroides; Indel, insert or deletion; ORF, open reading frame; ORFans, open reading frames of unknown functions; AlaRS, alanyl-tRNA synthetase; RGC, rare genetic change; RpoC, RNA polymerase β '-subunit.

## Authors' contributions

The initial blastp searches on various genomes were carried out by RSG with the computer assistance provided by Venus Wong. EL analyzed the results of these blast searches to identify various group-specific proteins and confirmed their specificities by means of PSI-blast and genomic blasts. RSG carried out the phylogenetic analyses and identified the conserved indels described here. RSG was also responsible for conceiving and directing this study and for the final evaluation of results. RSG was responsible for the preparation of the final manuscript. All authors have read and approved the submitted manuscript.

## Supplementary Material

Additional File 1 A conserved indel (3 aa deletion) in ClpB protein that is mainly specific for the *Bacteroidales*, *Flavobacteriales *and *Flexibacteraceae *species. Partial sequence alignment of the ClpB protein containing this indel region is shown. The boxed region is missing in the *Bacteroidales*, *Flavobacteriales *and *Flexibacteraceae *species. Dashes in the alignment show identity with the amino acid on the top line. The ClpB homologs from *C. phaebacteroidetes *and *Methanospirillum hungatei *also lack the boxed region, which could be due to LGT. The beta and gamma proteobacteria contain a larger insert in this region, which has likely occurred independently. Click here for file

Additional File 2 Proteins that are specific for the Bacteroides Genus. All significant hits for these proteins are from the following sequenced *Bacteroides *species unless otherwise indicated: *B. thetaiotaomicron *VPI-5482, *B. fragilis *NCTC 9343 and YCH46. Click here for file

Additional file 3 Proteins specific for Flavobacteria that are missing in several species. All significant hits for these proteins are also from *Flavobacteriale*s species. However, unlike the proteins listed in Table 5, these proteins are either present in only a small number of Flavobacteria or are missing from many species. Click here for file

Additional file 4 Chlorobi-specific proteins that are missing in some species. All significant hits for these proteins are also from *Chlorobi *species. However, unlike the proteins listed in Table 6, these proteins are not present in all *Chlorobi *species. Click here for file
