# Supplementary material for: Phylogeny and molecular signatures (conserved proteins and indels) that are specific for the Bacteroidetes and Chlorobi species
Source: BMC Evol Biol. 2007 May 8;7:71. doi: 10.1186/1471-2148-7-71 (PMC1887533; doi:10.1186/1471-2148-7-71)
Supplement: Additional File 1 — A conserved indel (3 aa deletion) in ClpB protein that is mainly specific for the Bacteroidales, Flavobacteriales and Flexibacteraceae species. Partial sequence alignment of the ClpB protein containing this indel region is shown. The boxed region is missing in the Bacteroidales, Flavobacteriales and Flexibacteraceae species. Dashes in the alignment show identity with the amino acid on the top line. The ClpB homologs from C. phaebacteroidetes and Methanospirillum hungatei also lack the boxed region, which could be due to LGT. The beta and gamma proteobacteria contain a larger insert in this region, which has likely occurred independently. [file 1471-2148-7-71-S1.pdf]

Additional File 1: A conserved indel (3 aa deletion) in ClpB protein that is mainly specific for the *Bacteroidales*, *Flavobacteriales* and *Flexibacteraceae* species

Group	Species	Accession	405		460
Bacteroidales, Flavobacteriales, Flexibacteraceae	Porphyromonas gingivalis	NP_905329	SLPEELDEISRRIKQLEIEREAIKRENDEEKVQFLDR		EIAELKEKEASEKAQWQNE
	Bact. fragilis	YP_210841	-V--G------K----------------P-L-TIGK		-L-----Q-K-Y--K--S-
	Bact. thetaiotaomicron	NP_813508	-V---------K---------------KP-LEIIGK		-L-----V-K-F--K--S-
	Algoriphagus sp. PR1	ZP_01721106	---Q----LN---M---------R--KNKD-ETV-SK		-L---A--RD-V--K-ES-
	Flavobacteria bacterium	ZP_01202344	-K-----VLD-K-M-----I------K--S-LKA-RS		-L-NF--SRNEIN---V--
	Croceibacter atlanticus	ZP_00950628	-K-----VLD-KVM-----I------K--V-LKS-RS		DL-N---ERNELH---K--
	Cellulophaga sp. MED134	ZP_01048884	-K-----VLD--VM-----I------K--D-LKS-RA		DL-N---ERNDLN----S-
	Flavo. johnsoniae	ZP_01243107	-K-----VLD-K-M-----I------KE-S-LKI-GM		-L-N---ERNEIY-K-KQ-
	Flavo. bacterium	ZP_01734788	-K-----VLD-K-M-----M---------T-LKG-GM		DL-N---ERNEIFTK-KS-
	Robiginitalea biformata	ZP_01121357	-K-----ALD-K-M-----I--------PD-LKA-NA		-L-NF--ERNEIF-K-ES-
	Psychroflexus torques	ZP_01254403	-K-----VLD-KVM-----I------K--T-LKS-RV		DL-D--DERNTLH-R-K--
	Flavobacteriales bacterium	ZP_01107726	-K-----VLD-K-M-----I------D-KA-LKL-NV		DL-NY---RNGVF-K-ES-
	Gramella forsetii	YP_862297	-K-----VLD-K-M-----I------K--A-LKS-RS		DL-N---ERNDLH-R-MS-
	Tenacibaculum sp. MED152	ZP_01051988	-K-----VLD-KVM-----I------K--V-LNS-RS		DL-N---ERNEIN-K-KS-
	Flavobacterium sp. MED217	ZP_01060964	-K-----VLD-K-M-----I------Q--N-LKS-RA		DL-N---ERNEIN-K-KS-
	Microscilla marina	ZP_01690660	-M-A----LQ-KVM---------R--KNKKREKEIS-		-----SQ-RD-L--R-ET-
	Cytophaga hutchinsonii	YP_679705	-M-Q-----L---M---------R---NK--ETI-S-		-L---N-QKSQIM-K--E-
Exceptions - other species containing this deletion	Cb. phaeobacteroides	ZP_00532807	------EIVE--VR------------H-QL-LDVISQ		-L-N-N-IRTDLR-R--A-
	Methanospirillum hungatei	YP_502751	-V----EI-E-K-R------------K-QV-LDA-NE		--GN-T-ERNRL--K--S-
	Salinibacter ruber	YP_446924	-M-AD--QLE-E-R--------V--DEGDQAEKLDEI	DR	K--D-EDERDEL--R-TE-
Proteobacteria	Brucella melitensis	NP_539113	-K-------D---M--K-----L-V-T-AASKDR-Q-	IEK	-LSD-E-ES-ELT-K--A-
	Rhodospirillum rubrum	YP_425844	-K--A---LD---I--K-----LRK-K-IASEAR-SD	LEK	-L-D-ESQS-TLTED-KR-
	Zymomonas mobilis	YP_163159	-K---IENLD---I--K-----LLK---DASRDR-AT	LEH	DLSD-EQQSSELTTR-K--
	Rickettsia rickettsii	ZP_00153127	-K------LD---I-IK--LA-L-K----HSKKKIEH	LTK	ALEK-ES-SYDMG-K--A-
	Paracoccus denitrificans	ZP_00630585	-K-----ALD-Q-L-MQ--A--L-K-D-AASQDR-EK	LEK	QLS--Q--S-TMT-R--A-
	Rhodopseudomonas palustris	YP_534120	-K-----SMD-E-IR-K--Q--L-K-T-PGSKSR-QT	LSG	-L---E--S-ALT-R-SA-
	Caulobacter crescentus	NP_419695	-K-------D--LV--K-----LSK-T-AASK-R-EN	LEV	--DD-QFRSDEMT-R-KA-
	Agrobacterium tumefaciens	NP_356471	-K------LD---I--K-----L-Q-T-QSS-DR-RK	LED	-L-DTE--ADALT-R--A-
	Rickettsia prowazekii	NP_220430	-K------LD---I-IK--LA-L-K----HSKKKITS	LTE	-LKK-ES-SYDMNTK--A-
	Des. desulfuricans	YP_388153	---A----AN-K-M--------LR--T-AASRER-QK	LEN	-L---R-VQ-GLNT--ER-
	Desulfovibrio vulgaris	NP_966736	---AD---AN-K-M--------LR--T-VASRER-E-	LEN	-L-D-RAEQTALLS--ER-
	Bdello. bacteriovorus	NP_969820	-V---V-K-E-ELM--R--K--L-K-K--SARER-AV	IDK	--T--NA-NQLLRE--EF-
	Myxococcus xanthus	YP_633246	-M-T---DVR-KMT--Q----GLRK-T-PHSQER-GQ	IEK	-L-N-S--FNAL-VH-DA-
	Geo. metallireducens	YP_385794	-M-T-I--VE-K-I-----KQ-LL--Q-PHALER-QK	LTD	-LNG-QAQA-EL--H-RQ-
Other Gram-ve and Gram+ve bacteria	C. Kuenenia stuttgartiens	CAJ71743	-M-V-----E-K-L-----K--L-K-K--ASK-RIEK	IER	QLSD---ESRAFR-H-E--
	Leptospira interrogans	YP_001955	-M-----RANK--QS-K-----L-K-Q-TASKER-KT	LER	DLS-QEQNFQTL--R-DL-
	Treponema pallidum	NP_218511	-Q-V---QVE-K-L--N--KASLLK-S-PASKER-EK	LEK	-L-GFL-RR-AMQV-----
	Chloroflexus aurantiacus	ZP_00768354	-D-Q---DLK---M--------LRK-K-QASKER-EK	LEQ	-L-N-R-QRSALE--I-R-
	Roseiflexus castenholzii	ZP_01531627	-D-Q---DLK---M--------L-K-K-KASKER-EK	LEQ	-L-N-Q-QRRAVE--L-R-
	Syn. elongatus PCC 6301	YP_171170	-K-------D-K-L---M--LSLQK-S-LASQER-Q-	LEK	-L-D---EQR-LSS---A-
	Synechocystis sp. PCC 6803	NP_441776	-K------VD-K-L---M--LSLQ----SASKER-EK	LEK	-L-DF--EQSKLNG---S-
	Nostoc sp. PCC 7120	NP_489124	-K-------D-K-L---M-KLSLQK-S-AASRER-E-	LEK	-L-D---EQRTLNT---S-
	Thermus thermophilus	YP_005092	-A---I-ALE-KKL--------L-K-K-PDSQER-KA	IEA	---K-T-EI-KLR-E-ER-
	Meiothermus ruber	Q7X2S8	-S--SI-ALN--KL--------L-K-T-A-SKFR-GE	LEK	---D-E-EIRKQQ-E-EA-
	Fuso. nucleatum	ZP_00143139	-M-----QLT-KAL-----IK-LQK-T-DASKER-KV	IEK	-L---N-EKKVLTSK-EL-
	Bacillus cereus	YP_082667	-M-T----VT---M-----EA-LGK-K-FGSQER-KT	LQR	-LSD---VAS-MR-K-EK-
	Lactococcus lactis	YP_001032307	---T---QAN--LM-----EA-L-K-R-DASKKR-EI	IRG	-----R-ENNQL----EA-
	Bac. anthracis	ZP_00391506	-M-T----VT---M-----EA-LGK-K-FGSQER-KT	LQR	-LSD---VAS-MR-K-EK-
	Listeria monocytogenes	EBA36322	-M-S----VT-KVM-----EA-L-E-K-PASERR-EI	LQR	-L-DY--EANQM-SK-ES-
	Enterococcus faecalis	NP_816010	-M-T---QVT--LM-----EA-L-K-S-DASKKR-AN	LQE	-L-D-R-EAN-M-M--ET-
	Coryne. glutamicum	NP_601973	-S-Q-I--LE-IVRR----EM-LSK-S-AASKER-EK	LRS	-L-DER--LSEL--R----
	Myc. Tuberculosis	NP_214898	-R-V-I--VE-LVRR----EM-LSK-E--ASAER-AK	LRS	-L-DQ---L-ELTTR----
	Cor. efficiens	NP_739223	-S-Q-I--LE-IVRR----EV-LTK-T-VASRER-E-	LRS	-L-DER--LSEL--R----
	Clo. tetani	NP_781219	---T---S-K-K-F-M---K--LAK-K-SRSKER-ED	LEK	-LSN----DKEMT-KYEK-
	Streptomyces avermitilis	NP_828417	-M-A-----T--VTR----EA-LSK-S-PASKTR-EE	LRR	-L-D-RGEADAKH---EA-
	Frankia sp. CcI3	YP_481876	-M-TG----T--VVR----EA-LAQ-S-PASIAR--Q	LRG	-L-DVRAAANTMR-R-EA-
Archaea	Methanococcoides burtonii	YP_566814	-K-S----AD-K-L--------L-K-K-AVSKERVAD	LEK	-L-DIRAESDAMR-K--S-
