# Supplementary material for: Phylogeny and molecular signatures (conserved proteins and indels) that are specific for the Bacteroidetes and Chlorobi species
Source: BMC Evol Biol. 2007 May 8;7:71. doi: 10.1186/1471-2148-7-71 (PMC1887533; doi:10.1186/1471-2148-7-71)
Supplement: Additional File 2 — Proteins that are specific for the Bacteroides Genus. All significant hits for these proteins are from the following sequenced Bacteroides species unless otherwise indicated: B. thetaiotaomicron VPI-5482, B. fragilis NCTC 9343 and YCH46. [file 1471-2148-7-71-S2.pdf]

Additional File 2: Proteins that are Specific for the Bacteroides Genus

Genome ID No. [Accession No.]	Possible/Predicted Function	Genome ID No. [Accession No.]	Possible/Predicted Function
BT0017 [NP_808930][*]	conserved hypothetical protein	BT1977 [NP_810890]	conserved hypothetical protein
BT0018 [NP_808931][*]	conserved hypothetical protein	BT1988 [NP_810901]	conserved hypothetical protein
BT0019 [NP_808932][*]	conserved hypothetical protein	BT2037 [NP_810950][*]	hypothetical protein
BT0020 [NP_808933][*]	conserved hypothetical protein	BT2057 [NP_810970]	conserved hypothetical protein
BT0080 [NP_808993][*]	conserved hypothetical protein	BT2083 [NP_810996][*]	conserved hypothetical protein
BT0081 [NP_808994]	conserved hypothetical protein	BT2125 [NP_811038]	putative lipoprotein
BT0088 [NP_809001]	Cons. protein maybe related to TraL; pfam00939, Na_sulph_symp	BT2170 [NP_811083]	putative exported protein
BT0092 [NP_809005]	conserved protein maybe related to TraH	BT2173 [NP_811086]	putative lipoprotein
BT0153 [NP_809066]	conserved hypothetical protein	BT2176 [NP_811089][*]	conserved hypothetical protein
BT0157 [NP_809070]	hypothetical protein	BT2178 [NP_811091]	putative exported protein
BT0177 [NP_809090]	putative lipoprotein	BT2215 [NP_811128]	putative transmembrane protein
BT0241 [NP_809154]	putative exported protein	BT2233 [NP_811146]	conserved hypothetical protein
BT0247 [NP_809160]	putative membrane protein	BT2283 [NP_811196][*]	conserved hypothetical protein
BT0255 [NP_809168]	conserved hypothetical protein; cd00291, SirA_YedF_YeeD	BT2284 [NP_811197][*]	conserved hypothetical protein
BT0256 [NP_809169]	conserved hypothetical protein	BT2286 [NP_811199]	conserved hypothetical protein
BT0293 [NP_809206]	putative exported protein; pfam06082, DUF940;	BT2291 [NP_811204]	conserved protein maybe related to TraL
BT0377 [NP_809290]	Putative transcriptional regulator; UpxZ homolgue	BT2312 [NP_811225]	hypothetical protein
BT0406 [NP_809319]	conserved hypothetical protein	BT2317 [NP_811230]	conserved hypothetical protein
BT0407 [NP_809320]	putative transmembrane protein	BT2337 [NP_811250]	conserved protein maybe related to TraH
BT0417 [NP_809330]	conserved hypothetical exported protein	BT2438 [NP_811351]	conserved hypothetical exported protein
BT0490 [NP_809403]	conserved membrane protein	BT2450 [NP_811363][*]	conserved hypothetical protein
BT0493 [NP_809406]	putative membrane protein	BT2466 [NP_811379][*]	conserved hypothetical protein
BT0498 [NP_809411]	putative lipoprotein	BT2470 [NP_811383][*]	putative outer membrane protein
BT0522 [NP_809435]	putative glycosyltransferase	BT2471 [NP_811384][*]	putative outer membrane protein
		BT2500 [NP_811413]	put. exported protein
BT0593 [NP_809506]	conserved hypothetical protein	BT2503 [NP_811416]	hypothetical protein
BT0597 [NP_809510]	Putative transcriptional regulator; UpxZ homolgue	BT2504 [NP_811417]	conserved hypothetical exported protein
BT0641 [NP_809554]	putative lipoprotein	BT2535 [NP_811448][*]	conserved hypothetical protein
BT0659 [NP_809572]	putative exported protein	BT2545 [NP_811458]	putative transmembrane protein
BT0661 [NP_809574]	putative exported protein	BT2601 [NP_811514]	conserved protein maybe related to TraH
BT0664 [NP_809577]	putative exported protein	BT2657 [NP_811570]	putative exported protein
BT0666 [NP_809579]	putative exported protein	BT2658 [NP_811571]	putative lipoprotein
BT0713 [NP_809626]	hypothetical membrane protein	BT2669 [NP_811582]	conserved hypothetical protein
BT0723 [NP_809636][*]	hypothetical protein	BT2695 [NP_811607]	conserved hypothetical protein
BT0731 [NP_809644]	putative exported protein	BT2698 [NP_811610]	putative transmembrane protein
BT0777 [NP_809690]	putative transmembrane protein	BT2699 [NP_811611]	conserved hypothetical protein
BT0809 [NP_809722][+]	putative lipoprotein	BT2848 [NP_811760]	putative lipoprotein
BT0830 [NP_809743]	hypothetical protein	BT2982 [NP_811894][*]	conserved hypothetical protein
BT0880 [NP_809793]	conserved hypothetical protein	BT3056 [NP_811968]	putative lipoprotein
BT0881 [NP_809794]	putative exported protein	BT3059 [NP_811971]	conserved hypothetical protein
BT0950 [NP_809863][*]	conserved hypothetical protein	BT3185 [NP_812097]	hypothetical transmembrane protein; pfam06713, DUF1200
BT1038 [NP_809951]	putative secreted endoglycosidase	BT3217 [NP_812129][*]	hypothetical protein
BT1044 [NP_809957]	putative secreted endoglycosidase; pfam00704, Glyco_hydro_18	BT3252 [NP_812164][+]	putative transmembrane protein
BT1065 [NP_809978][*]	conserved hypothetical protein	BT3259 [NP_812171]	putative exported protein
BT1067 [NP_809980]	putative exported protein	BT3323 [NP_812235]	putative exported protein
BT1071 [NP_809984]	hypothetical protein	BT3400 [NP_812312]	putative transmembrane protein
BT1072 [NP_809985]	hypothetical protein	BT3541 [NP_812453]	hypothetical protein
BT1074 [NP_809987]	conserved hypothetical exported protein	BT3629 [NP_812540]	putative lipoprotein
BT1080 [NP_809993][+]	putative transmembrane protein	BT3715 [NP_812626][+]	hypothetical protein
BT1140 [NP_810053]	conserved hypothetical protein	BT3721 [NP_812632]	conserved hypothetical protein
BT1142 [NP_810055]	hypothetical protein	BT3727 [NP_812638]	conserved hypothetical protein
BT1170 [NP_810083]	conserved hypothetical protein	BT3737 [NP_812648]	hypothetical protein
BT1182 [NP_810095]	conserved hypothetical protein	BT3747 [NP_812658]	conserved hypothetical protein

BT1282 [NP_810195]	putative secreted endoglycosidase	BT3753 [NP_812664]	putative secreted endoglycosidase; pfam00704, Glyco_hydro_18; smart00636, Glyco_18
BT1287 [NP_810200]	putative exported protein	BT3822 [NP_812733]	putative transmembrane protein
BT1303 [NP_810216]	conserved hypothetical protein	BT3856 [NP_812767]	putative exported protein
BT1305 [NP_810218]	putative transmembrane protein	BT3874 [NP_812785]	putative exported protein
BT1309 [NP_810222]	conserved exported hypothetical protein	BT3886 [NP_812797]	conserved hypothetical protein
BT1357 [NP_810270]	Putative transcriptional regulator; UpxZ homolgue	BT3949 [NP_812860]	putative exported protein
BT1366 [NP_810279]	putative conserved exported protein	BT3976 [NP_812887]	putative transmembrane protein
BT1369 [NP_810282]	conserved hypothetical protein	BT3985 [NP_812896]	Putative secreted endoglycosidase
BT1421 [NP_810334]	hypothetical membrane protein	BT4005 [NP_812916]	hypothetical protein
BT1435 [NP_810348]	hypothetical protein	BT4019 [NP_812930]*	conserved hypothetical protein
BT1461 [NP_810374]*	hypothetical protein	BT4068 [NP_812979]	conserved hypothetical protein
BT1462 [NP_810375]	putative membrane protein	BT4196 [NP_813107]	putative transmembrane protein
BT1480 [NP_810393]	conserved hypothetical protein	BT4219 [NP_813130]+	hypothetical protein
BT1556 [NP_810469]	conserved hypothetical protein	BT4226 [NP_813137]	putative exported protein
BT1557 [NP_810470]	conserved hypothetical protein	BT4261[NP_813172]	hypothetical protein
BT1558 [NP_810471]	RNA polymerase ECF-type sigma factor	BT4283 [NP_813194]	hypothetical protein
BT1561 [NP_810474]	conserved hypothetical exported protein	BT4325 [NP_813236]	Putative ATP/GTP-binding transmembrane protein; COG2177, FtsX
BT1579 [NP_810492]	conserved hypothetical protein	BT4327 [NP_813238]	conserved hypothetical protein
BT1589 [NP_810502]	possible cell division protein	BT4348 [NP_813259]+	hypothetical protein
BT1611 [NP_810524]*	conserved hypothetical protein	BT4381 [NP_813292]	putative membrane protein
BT1612 [NP_810525]+	putative membrane protein	BT4413 [NP_813324]	hypothetical protein
BT1614 [NP_810527]	putative membrane protein	BT4414 [NP_813325]	conserved hypothetical protein
BT1639 [NP_810552]	conserved hypothetical protein	BT4431 [NP_813342]*	conserved hypothetical protein
BT1655 [NP_810568]	Putative transcriptional regulator; UpxZ homolgue	BT4467 [NP_813378]*	conserved hypothetical protein
BT1679 [NP_810592]	putative membrane protein	BT4475 [NP_813386]	conserved hypothetical protein
BT1698 [NP_810611]	putative membrane protein; COG3630.2, OadG	BT4498 [NP_813409]	conserved hypothetical protein
BT1704 [NP_810617]	conserved hypothetical protein	BT4505 [NP_813416]	conserved hypothetical protein
BT1724 [NP_810637]	Putative transcriptional regulator; UpxZ homolgue	BT4527 [NP_813438]*	conserved hypothetical protein
BT1740 [NP_810653]+	putative exported protein	BT4563 [NP_813474]	hypothetical protein
BT1741 [NP_810654]	hypothetical membrane protein	BT4569 [NP_813480]	putative transmembrane protein
BT1784 [NP_810697]	conserved hypothetical protein	BT4570 [NP_813481]	putative transmembrane protein
BT1816 [NP_810729]	conserved hypothetical protein	BT4596 [NP_813507]	conserved hypothetical protein
BT1819 [NP_810732]	conserved hypothetical protein	BT4598 [NP_813509]	conserved hypothetical protein
BT1895 [NP_810808]	conserved hypothetical protein	BT4606 [NP_813517]	putative lipoprotein
BT1908 [NP_810821]	putative exported protein	BT4613 [NP_813524]	conserved hypothetical protein
BT1925 [NP_810838]	hypothetical protein	BT4640 [NP_813551]	hypothetical protein
BT1926 [NP_810839]	putative exported protein	BT4674 [NP_813585]	hypothetical protein
BT1946 [NP_810859]*	conserved hypothetical protein	BT4685 [NP_813596]	putative exported protein
BT1947 [NP_810860]*	conserved hypothetical protein	BT4719 [NP_813630]	hypothetical protein
BT1959 [NP_810872]+	putative phage integrase	BT4721 [NP_813632]	RNA Pol. ECF-type sigma factor
BT1963 [NP_810876]+	putative transcriptional regulator		

All significant hits for theses proteins are from the following sequenced *Bacteroides* species unless otherwise indicated: *B. thetaiotaomicron* VPI-5482, *B. fragilis* NCTC 9343 and YCH46.

*Missing in one of the *B. fragilis* strains.
+Significant similarity also seen for 1 or more Prevotella species.

The following proteins in this Table are present in gene clusters and could be involved in related cellular functions: (BT0017-BT0020, BT0080-BT0081, BT0406-BT0407, BT0880-BT0881, BT1071-BT1072, BT1461-BT1462, BT1556-BT1558, BT1611-BT1612, BT1740-BT1741, BT1925-BT1926, BT1946-BT1947, BT2283-BT2284, BT2470-BT2471, BT2503-BT2504, BT2657-BT2658, BT2698-BT2699, BT4413-BT4414, BT4569-BT4570)

The following proteins in this Table are homologous to each other and have resulted from gene duplication events.
BT0088, BT2291; BT0092, BT2601, BT2337; BT0157, BT1072; BT0377, BT0597, BT1357, BT1655, BT1724; BT0661, BT0664, BT0666; BT0830, BT4674; BT0950, BT4431; BT1038, BT1044, BT3753, BT3985; BT1071, BT2312; BT1074, BT2438; BT1142, BT2503, BT2504; BT1170, BT1639, BT1704, BT1988; BT1435, BT3737; BT1461, BT2535; BT1558, BT4721; BT2317, BT3059; BT2500, BT3259; BT2657, BT4226; BT2698, BT3822; BT4005, BT4719; BT4413, BT4640.
