# Supplementary material for: Phylogeny and molecular signatures (conserved proteins and indels) that are specific for the Bacteroidetes and Chlorobi species
Source: BMC Evol Biol. 2007 May 8;7:71. doi: 10.1186/1471-2148-7-71 (PMC1887533; doi:10.1186/1471-2148-7-71)
Supplement: Additional file 3 — Proteins specific for Flavobacteria that are missing in several species. All significant hits for these proteins are also from Flavobacteriales species. However, unlike the proteins listed in Table 5, these proteins are either present in only a small number of Flavobacteria or are missing from many species. [file 1471-2148-7-71-S3.pdf]

Additional File 3: Proteins Specific for Flavobacteria that are missing in several species

orf No. [Accession No.]	Possible/Predicted Function	Genome ID No. [Accession No.]	Possible/Predicted Function
orf31 [CAL65021]	hypothetical protein	orf1584 [CAL66570]	secreted protein
orf34 [CAL65024]	secreted protein	orf1661 [CAL66647]	hypothetical protein
orf48 [CAL65037]	secreted protein	orf1755 [CAL66741]	hypothetical protein
orf67 [CAL65056]	conserved hypothetical protein	orf1764 [CAL66750]	membrane protein
orf137 [CAL65126]	secreted protein	orf1769 [CAL66755]	hypothetical protein
orf150 [CAL65139]	hypothetical protein	orf1790 [CAL66776]	hypothetical protein
orf177 [CAL65166]	membrane protein	orf1798 [CAL66784]	hypothetical protein
orf231 [CAL65220]	hypothetical protein	orf1848 [CAL66834]	membrane or secreted protein
orf246 [CAL65235]	membrane protein	orf1855 [CAL66841]	secreted protein
orf249 [CAL65238]	hypothetical protein	orf1868 [CAL66854]	membrane protein
orf259 [CAL65248]	hypothetical protein	orf1869 [CAL66855]	membrane protein
orf341 [CAL65330]	secreted protein	orf1876 [CAL66862]	hypothetical protein
orf332 [CAL65321]	secreted protein	orf1903 [CAL66887]	hypothetical protein
orf347 [CAL65336]	membrane protein	orf1912 [CAL66896]	hypothetical; COG0626, MetC
orf459 [CAL65448]	hypothetical protein	orf2042 [CAL67026]	membrane or secreted protein
orf470 [CAL65459]	membrane protein	orf2160 [CAL67144]	hypothetical protein
orf467 [CAL65456]	membrane protein	orf2183 [CAL67167]	hypothetical; DUF704, Aha1 domain
orf479 [CAL65468]	hypothetical protein	orf2193 [CAL67177]	secreted protein
orf484 [CAL65473]	hypothetical protein	orf2223 [CAL67207]	hypothetical protein
orf503 [CAL65492]	conserved hypothetical protein, secreted	orf2289 [CAL67273]	hypothetical protein
orf520 [CAL65509]	membrane protein	orf2317 [CAL67301]	membrane protein
orf557 [CAL65546]	hypothetical protein	orf2318 [CAL67302]	hypothetical protein
orf568 [CAL65557]	Hypothetical protein-possible UDP-glycosyltransferase; COG0707, MurG	orf2483 [CAL67467]	hypothetical protein
orf603 [CAL65590]	hypothetical protein	orf2531 [CAL67515]	hypothetical protein
orf604 [CAL65591]	membrane protein	orf2548 [CAL67532]	hypothetical protein
orf663 [CAL65650]	hypothetical protein	orf2553 [CAL67537]	hypothetical protein
orf697 [CAL65684]	hypothetical protein	orf2611 [CAL67595]	membrane protein
orf720 [CAL65707]	hypothetical protein	orf2632 [CAL67616]	conserved hypothetical protein, membrane
orf781 [CAL65768]	hypothetical protein	orf2635 [CAL67619]	secreted protein
orf825 [CAL65812]	hypothetical protein	orf2672 [CAL67656]	membrane or secreted protein
orf844 [CAL65831]	secreted protein	orf2685 [CAL67669]	hypothetical protein
orf911 [CAL65898]	secreted protein	orf2729 [CAL67713]	secreted protein; COG2367, PenP
orf944 [CAL65931]	hypothetical protein	orf2733 [CAL67717]	membrane protein
orf969 [CAL65956]	secreted protein	orf2770 [CAL67754]	hypothetical protein
orf970 [CAL65957]	secreted protein	orf2772 [CAL67756]	membrane protein
orf971 [CAL65958]	secreted protein	orf2781 [CAL67765]	secreted protein
orf989 [CAL65976]	secreted protein	orf2827 [CAL67811]	secreted protein
orf1080 [CAL66067]	hypothetical protein	orf2843 [CAL67827]	hypothetical protein
orf1045 [CAL66032]	hypothetical protein	orf2904 [CAL67886]	hypothetical protein
orf1049 [CAL66036]	membrane protein	orf2909 [CAL67891]	hypothetical protein
orf1054 [CAL66041]	hypothetical protein	orf2918 [CAL67900]	membrane protein
orf1063 [CAL66050]	hypothetical protein	orf2930 [CAL67912]	membrane protein
orf1120 [CAL66107]	hypothetical protein	orf2960 [CAL67942]	secreted protein
orf1153 [CAL66140]	secreted protein	orf3011 [CAL67993]	secreted protein; cd03467, Rieske
orf1156 [CAL66143]	copper resistance protein A precursor	orf3054 [CAL68036]	membrane protein
orf1159 [CAL66146]	membrane or secreted protein	orf3067 [CAL68049]	hypothetical protein
orf1172 [CAL66159]	hypothetical protein	orf3075 [CAL68057]	secreted protein
orf1177 [CAL66167]	hypothetical protein	orf3084 [CAL68066]	membrane or secreted protein
orf1201 [CAL66188]	membrane protein	orf3090 [CAL68072]	hypothetical protein
orf1223 [CAL66210]	secreted protein	orf3095 [CAL68077]	hypothetical protein
orf1225 [CAL66212]	hypothetical protein	orf3101 [CAL68083]	secreted protein
orf1228 [CAL66215]	hypothetical protein	orf3143 [CAL68125]	secreted protein
orf1237 [CAL66224]	secreted protein	orf3149 [CAL68131]	secreted protein; COG3047, OmpW

orf1302 [CAL66289]	secreted protein	orf3188 [CAL68170]	hypothetical protein
orf1304 [CAL66291]	secreted protein	orf3207 [CAL68190]	secreted protein
orf1314 [CAL66301]	secreted protein	orf3215 [CAL68198]	secreted protein
orf1341 [CAL66328]	hypothetical protein	orf3216 [CAL68199]	hypothetical protein
orf1346 [CAL66333]	membrane or secreted protein	orf3221 [CAL68204]	hypothetical protein
orf1350 [CAL66337]	conserved hypothetical protein, membrane	orf3224 [CAL68207]	membrane or secreted protein
orf1354 [CAL66341]	membrane protein	orf3265 [CAL68248]	membrane protein
orf1374 [CAL66361]	Transposase 8, pfam01527	orf3344 [CAL68327]	secreted protein
orf1410 [CAL66397]	membrane protein	orf3380 [CAL68363]	membrane protein
orf1413 [CAL66400]	secreted protein	orf3385 [CAL68368]	secreted protein
orf1417 [CAL66404]	membrane or secreted protein	orf3407 [CAL68390]	secreted protein
orf1419 [CAL66406]	cbb3-type cytochrome oxidase maturation protein; COG3197, FixS	orf3410 [CAL68393]	secreted protein
orf1421 [CAL66408]	hypothetical protein	orf3436 [CAL68419]	membrane protein
orf1437 [CAL66424]	hypothetical protein	orf3449 [CAL68432]	membrane protein
orf1451 [CAL66438]	secreted protein	orf3450 [CAL68433]	membrane protein
orf1487 [CAL66474]	hypothetical protein	orf3477 [CAL68460]	hypothetical protein
orf1510 [CAL66497]	hypothetical protein	orf3540 [CAL68523]	conserved hypothetical protein, membrane
orf1582 [CAL66569]	hypothetical protein	orf3577 [CAL68560]	secreted protein

All significant hits for these proteins are also from *Flavobacteriale*s species. However, unlike the proteins listed in Table 5, these proteins are either present in only a small number of Flavobacteria or are missing from many species.

Of the proteins listed in this table and Table 5, the following proteins are homologous to each other: CAL67682, CAL67684; CAL67899, CAL66301, CAL66210; CAL65021, CAL66159; CAL65056, CAL65220; CAL65898, CAL66289; CAL66140, CAL66212, CAL66215, CAL67886, CAL67891; CAL65459, CAL65456; CAL66647, CAL66887; CAL65957, CAL65958.
