# Supplementary material for: Phylogeny and molecular signatures (conserved proteins and indels) that are specific for the Bacteroidetes and Chlorobi species
Source: BMC Evol Biol. 2007 May 8;7:71. doi: 10.1186/1471-2148-7-71 (PMC1887533; doi:10.1186/1471-2148-7-71)
Supplement: Additional file 4 — Chlorobi-specific proteins that are missing in some species. All significant hits for these proteins are also from Chlorobi species. However, unlike the proteins listed in Table 6, these proteins are not present in all Chlorobi species. [file 1471-2148-7-71-S4.pdf]

Additional File 4: *Chlorobi*-Specific Proteins that are Missing in Some Species

Genome ID No. [Accession No.]	Possible/Predicted Function	Genome ID No. [Accession No.]	Possible/Predicted Function
Plut_0107 [YP_374040]	Hypothetical protein	Plut_1429 [YP_375331]	Hypothetical protein
Plut_0759 [YP_374677]	Hypothetical protein	Plut_1673 [YP_375570]	Hypothetical protein
Plut_0762 [YP_374678]	Hypothetical protein	Plut_1729 [YP_375626]	Hypothetical protein
Plut_0981 [YP_374886]	Hypothetical protein	Plut_1790 [YP_375687]	Hypothetical protein
Plut_0985 [YP_374890]	Hypothetical protein	Plut_1807 [YP_375704]	Hypothetical protein
Plut_1092 [YP_374997]	Hypothetical protein	Plut_1865 [YP_375756]	Hypothetical protein
Plut_1145 [YP_375050]	Hypothetical protein	Plut_1870 [YP_375761]	Hypothetical protein
Plut_1858 [YP_375751]	Hypothetical protein	Plut_1953 [YP_375838]	Hypothetical protein
Plut_0072 [YP_374005]	Hypothetical protein	Plut_2004 [YP_375889]	Hypothetical protein
Plut_0081 [YP_374014]	Hypothetical protein	Plut_2099 [YP_375984]	Hypothetical protein
Plut_0159 [YP_374092]	Hypothetical protein	Plut_2102 [YP_375987]	Hypothetical protein
Plut_0232 [YP_374163]	Hypothetical protein	CT0071 [NP_660977]	Hypothetical; COG1729
Plut_0248 [YP_374179]	Hypothetical protein	CT0222 [NP_661127]	Hypothetical protein
Plut_0341 [YP_374272]	Hypothetical protein	CT0419 [NP_661323]	TonB-dependent receptor
Plut_0415 [YP_374346]	Hypothetical; COG2250; DUF712, pfam05168	CT0686 [NP_661582]	Hypothetical protein
Plut_0426 [YP_374357]	Hypothetical protein	CT0793 [NP_661688]	Hypothetical protein
Plut_0470 [YP_374401]	Hypothetical protein	CT1002 [NP_661895]	Hypothetical protein
Plut_0524 [YP_374451]	Hypothetical protein	CT1004 [NP_661897]	Hypothetical protein
Plut_0563 [YP_374489]	Hypothetical protein	CT1065 [NP_661956]	Hypothetical protein
Plut_0758 [YP_374676]	Hypothetical protein	CT1122 [NP_662013]	Hypothetical protein
Plut_0852 [YP_374761]	Transposase, IS4	CT1217 [NP_662107]	Hypothetical protein
Plut_0865 [YP_374773]	Hypothetical protein	CT1238 [NP_662126]	Hypothetical protein
Plut_0974 [YP_374879]	Hypothetical protein	CT1273 [NP_662161]	Hypothetical protein
Plut_0978 [YP_374883]	Hypothetical; YfaZ precursor, pfam07437	CT1380 [NP_662267]	Hypothetical protein
Plut_0989 [YP_374894]	Hypothetical protein	CT1504 [NP_662389]	Hypothetical protein
Plut_1095 [YP_375000]	Hypothetical protein	CT1621 [NP_662504]	Hypothetical protein
Plut_1103 [YP_375008]	Hypothetical protein	CT1658 [NP_662541]	Hypothetical protein
Plut_1249 [YP_375154]	Hypothetical protein	CT1739 [NP_662619]	Hypothetical protein
Plut_1256 [YP_375161]	Hypothetical protein	CT1801 [NP_662680]	Hypothetical protein; pfam06727, DUF1207
Plut_1268 [YP_375173]	Sodium pump decarboxylase, gamma subunit	CT1843 [NP_662721]	Hypothetical protein
Plut_1345 [YP_375247][*]	Hypothetical protein	CT1889 [NP_662766]	Hypothetical protein
Plut_1362 [YP_375264]	Glycosyl transferase WecB/TagA/CpsF	CT2182 [NP_663055]	ribosomal protein L29; pfam00831
Plut_1374 [YP_375276]	Hypothetical protein		

All significant hits for these proteins are also from *Chlorobi* species. However, unlike the proteins listed in Table 6, these proteins are not present in all *Chlorobi* species. For the protein Plut_1345, a homolog showing significant similarity is also found in *Cytophaga hutchinsonii*.
The first 8 proteins (viz. Plut_0107, Plut_0759, Plut_0762, Plut_0981, Plut_0985, Plut_1092, Plut_1145, Plut_1858) are unique to only *C. luteolum* DSM 273 and *C. phaeovibrioides* DSM 265.
